# Exploiting SpyTag/SpyCatcher Technology to Design New Artificial Catalytic Copper Proteins

**DOI:** 10.1002/cbic.202500208

**Published:** 2025-05-21

**Authors:** Silvia Gentili, Francesca Miglioli, Valentina Borghesani, Gloria Spagnoli, Denise Bellotti, Davide Cavazzini, Remo Guerrini, Maurizio Remelli, Giovanni Maestri, Simone Ottonello, Angelo Bolchi, Matteo Tegoni

**Affiliations:** ^1^ Department of Chemistry, Life Sciences, and Environmental Sustainability University of Parma Parco Area delle Scienze 11A 43124 Parma Italy; ^2^ Department of Chemical, Pharmaceutical and Agricultural Sciences University of Ferrara 44100 Ferrara Italy

**Keywords:** artificial metalloproteins, ATCUN, copper proteins, protein design, spy protein

## Abstract

Designing artificial metal binding sites within a protein is challenging since amino acid residues need to be placed in desired positions in the final construct and the use of non‐natural amino acids is difficult. The alternative approach of directing the insertion of artificial metal coordination systems presents the difficulty of grafting such site in a single desired position. Spy protein is composed of a protein component (SpyCatcher) which binds spontaneously an oligopeptide (SpyTag) with formation of an isopeptide bond. A SpyTag peptide equipped with an ATCUN (amino terminal copper and nickel) binding site is designed to bind copper(II) with high femtomolar affinity both in the absence of SpyCatcher and in the reconstituted Spy construct. The Cu^2+^ ATCUN site in the reconstituted Spy protein presents a catalytic activity in reactive oxygen species production, higher than that of the SpyTag peptide alone. This method offers a novel approach for constructing artificial metalloproteins by incorporating functional metal binding sites into a peptide, which can then be clicked onto its protein counterpart. The small size and modularity of this construct make it versatile for integration into other protein systems, eventually moving the complexity from a protein to a peptide and highlighting its potential for protein design.

## Graphical abstract

Designing artificial metal sites in proteins is challenging due to the need to place the metal site in precise positions and to tailor the coordination environment of the metal. A modular approach based on the SpyTag/SpyCatcher technology is used to provide the Spy construct with a Cu^2+^/ATCUN site. This strategy enables versatile metalloprotein design by shifting complexity from a protein to a peptide.

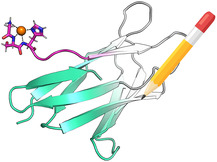

## 1 Introduction

Metalloproteins promote a number of the most complex natural biomolecular processes. Protein design is a powerful tool for the creation of tailored proteins that either do not exist or have different functions compared to those present in Nature. In particular, metalloprotein design has become the target of a tremendous effort, which has led to the development of protein constructs bearing mimics of biologically relevant metal binding sites capable of recreating their function and, most excitingly, achieving new ones.^[^ ^1^, ^2^, ^3^, ^4^ ^]^

The increasing demand for synthetic catalysts with enzyme‐like properties is moving toward the development of ARTificial metalloenZYMES (ARTZYMES or ArMs).^[^ ^5^, ^6^, ^7^, ^8^, ^9^, ^10^ ^]^ Protein redesign and de novo design are the two major strategies for the development of Artzymes.^[^ ^1^, ^11^, ^12^, ^13^ ^]^ While the former focuses on the rational or combinatorial introduction of mutations in a known protein sequence,^[^ ^14^, ^15^, ^16^, ^17^, ^18^, ^19^, ^20^ ^]^ the latter aims at the development of relatively short peptides that assemble into minimalistic protein‐like constructs.^[^ ^1^, ^21^, ^22^, ^23^, ^24^ ^]^ Both strategies have potentialities and drawbacks related, in particular, to the introduction of metal binding sites into a given construct. *De novo* design allows a more straightforward construction of metal binding sites with specific donor atoms in its first coordination sphere.^[^ ^25^, ^26^, ^27^, ^28^ ^]^ This strategy often takes advantage of the use of amino acids with non‐natural side chains.^[^ ^1^ ^]^ On the other hand, the redesign of natural proteins may allow to equip metal centers with a more complex second coordination sphere.^[^ ^29^ ^]^ However, this comes together with the inherent difficulty associated with the introduction of functional artificial metal binding sites onto a natural protein scaffold.^[^ ^6^, ^14^ ^]^

In this work, we present a new approach for the design and construction of artificial metalloproteins that is reminiscent of the so‐called *Trojan‐horse* strategy.^[^ ^30^, ^31^, ^32^, ^33^ ^]^ A compelling example of this approach is the biotin/streptavidin technology pioneered by Ward and colleagues, which relies on metal coordination systems covalently bound to biotin (*horse*) that are taken inside the streptavidin protein pocket (*city of Troy*) as a consequence of the strong biotin–streptavidin interaction.^[^ ^17^, ^19^, ^34^, ^35^, ^36^ ^]^

**Figure 1:**
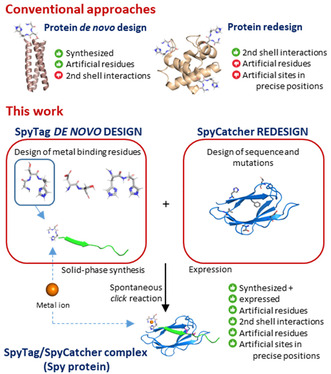
Above: Conceptual representation of the *Trojan horse* approach in artificial metalloprotein design. Below: representation of the complex formation between SpyTag (magenta) and SpyCatcher (blue) through formation of an isopeptide bond between Asp7 of SpyTag and Lys31 of SpyCatcher.

Herein, we describe an original design in which the *horse* is instead an oligopeptide (**Figure** **1**). This may offer three main advantages compared to other redesign approaches. First, an oligopeptide can be provided with metal‐binding sites for various soft and hard metal ions by a wise selection of just a few amino acid residues within a given core peptide sequence. Furthermore, oligopeptides can be easily synthesized and do not necessarily need to be of recombinantly expressed, even with the incorporation of non‐natural amino acids, granting a much more streamlined purification and characterization. Finally, oligopeptides are relatively small molecules that still retain a sufficient degree of complexity to permit their functionalization with more than one metal‐binding site on a single sequence. If the final artificial peptide can be clicked onto a protein, a construct with artificial functional metal binding sites can be obtained. Through this strategy, we shifted the complexity of controlling the introduction of metal binding sites on a protein from a relatively large protein to a relatively small peptide.

**Figure 2:**
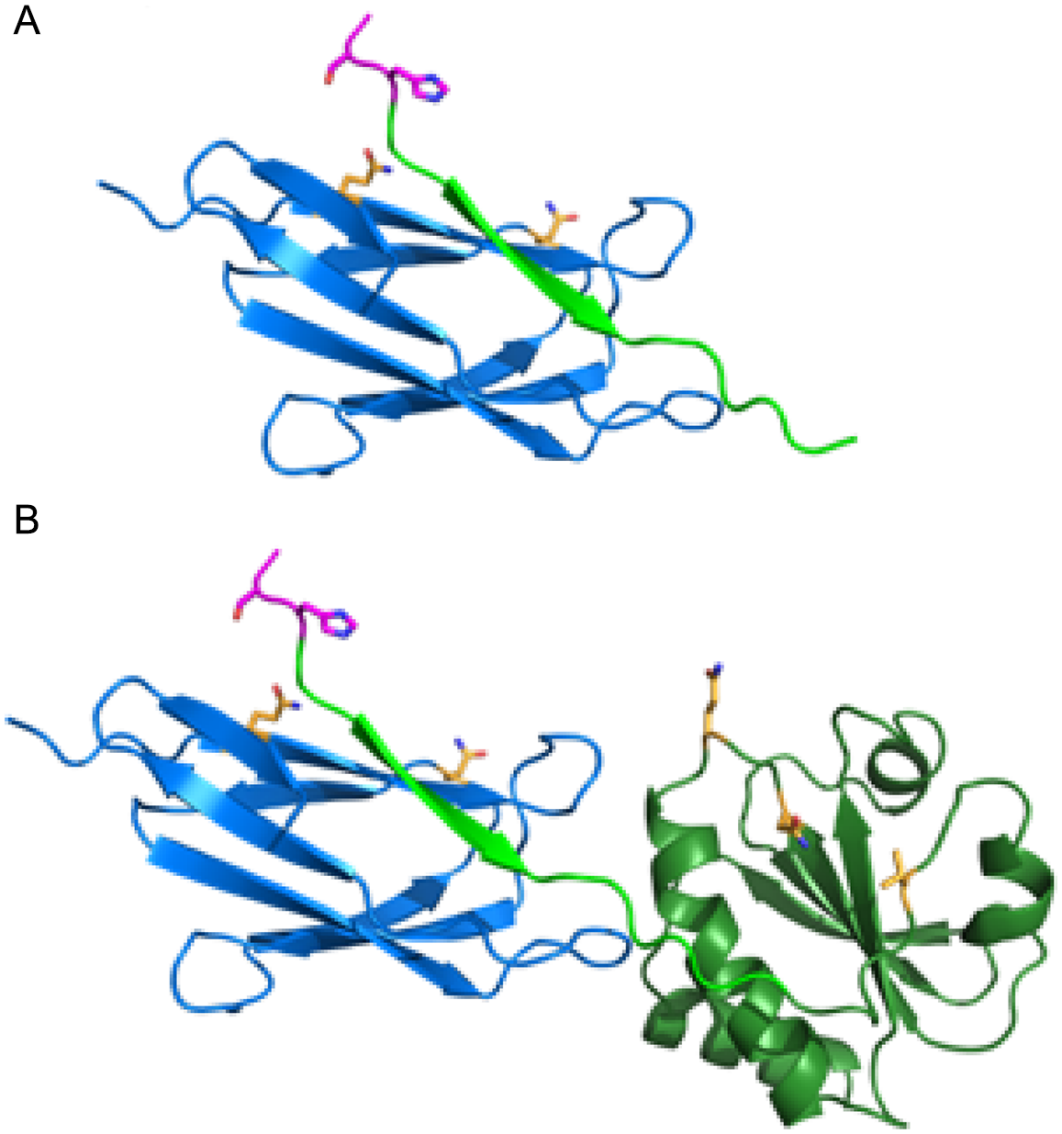
Representation of model structures of the A) **ST‐A1/SC1m** and B) **ST‐A3/SC2m** constructs obtained with AlphaFold2. The SpyTag peptide is in light green, with the ATCUN site in cornflower blue. The SpyCatcher domain is in blue with the two mutated His residues (H26Q and H62Q) represented in orange. The nonstructured N‐terminal segments of 32 and 26 residues of **SC1m** and of **SC2m** SpyCatcher are omitted for the sake of clarity. The *E. coli* thioredoxin domain is in dark green with the three mutated His residues represented in orange.

The Spy protein redesigned by Howarth and colleagues^[^ ^37^, ^38^, ^39^, ^40^ ^]^ may be instrumental to this goal (Figure 1). Spy is comprised of two components: the beta‐sandwich SpyCatcher (SC) protein and the SpyTag (ST) peptide.^[^ ^37^, ^38^, ^39^, ^40^ ^]^ SpyCatcher is a small 113 residues protein, while the SpyTag is an oligopeptide (or a segment of a protein) that includes the 11 residues sequence IVMVDAYKRYK (**Table** **1** and Table S1, Supporting Information). When mixed in solution, SC and ST interact rapidly and selectively to form an isopeptide bond between a Lys and an Asp residues located on the Catcher and the Tag components, respectively.^[^ ^37^, ^38^, ^39^, ^40^ ^]^ SC/ST complex formation is accomplished through an autocatalytic *click* reaction that occurs in buffered aqueous solutions at neutral pH within a few minutes, enabling precise ligation of the two components in a 1:1 ratio without the need for activating dyes.^[^ ^37^, ^38^, ^39^, ^40^ ^]^ For this reaction to occur, ST has to bear the consensus IVMVD peptide fragment that is responsible for the *lock‐and‐key* interaction with SC, with the Asp residue within the ST sequence being the one involved in isopeptide bond formation.^[^ ^37^, ^38^, ^39^, ^40^ ^]^ The crystal structure of the ST/SC complex shows that the adduct is a beta‐sandwich (**Figure** **2** and PDB ID 4MLI), while the first 20 residues of the SC are likely nonstructured (Figure S1A, Supporting Information).

**Table 1:** Amino acid sequences of SpyCatcher proteins and SpyTag peptides.

Proteins used in this work	Short names	Sequences	Notesa)
SpyCatcher002‐mut (H26Q H62Q)	SC1m	SGLVPRGSGAM^1^VTTLSGLSGEQGPSGDMTTEE*DSAT* ***Q*** *IKFSKRDEDGRELAGATMELRD‐ SSGKTISTWISDG**Q**VKDFYLYPGKYTFVETAAPDGYEVATAITFTVNEQGQVTVN*	Cleaved with TEV protease
	SC2m	‐‐‐‐‐‐‐GSGAM^1^VTTLSGLSGEQGPSGDMTTEE*DSAT**Q**IKFSKRDEDGRELAGATMELRD‐ SSGKTISTWISDG**Q**VKDFYLYPGKYTFVETAAPDGYEVATAITFTVNEQGQVTVN*	Cleaved with thrombin
			
ATCUN(GSH)‐SpyTag002	ST‐A1	GSHVPT**I** **V** **M** **V** **D**AYKRYK	
ATCUN(DAH)‐SpyTag002	ST‐A2	DAHVPT**IVMV** **D**AYKRY‐Am	
ATCUN(GSH)‐SpyTag002‐EcTrx	ST‐A3	GSHVPT**I** **V** **M** **V** **D**AYKRYK*SDKII**V**LTDDSFDTDVLKADGAILVDF**N**AE**Q**CGPCKMIAPILDEIADE‐YQGKLTVAKLNIDQNPGTAPKYGIRGIPTLLLFKNGEVAATKVGALSKGQLKEFLDANLA*	

a) The conserved sequence of SpyTag required for covalent binding to SpyCatcher is in bold. The Asp (D) residues that are involved in isopeptide bond formation are highlighted in bold and underlined. The structured domain of proteins is represented in italics. Mutated residues with respect to the literature sequences of proteins are shown in bold. The ATCUN sites are underlined. Sequence numbering follows that reported in ref. [39].

The Spy system has found a variety of applications since its first description in 2012, including the generation of specifically designed fusion protein complexes and vaccine production.^[^ ^38^, ^41^, ^42^, ^43^, ^44^, ^45^ ^]^ However, reports on the use of the Spy system to develop new metalloenzymes are very scarce and limited to the construction of fusion proteins or metallobiopolymers.^[^ ^46^, ^47^, ^48^, ^49^ ^]^

In this study, we describe the incorporation of a transition‐metal binding site onto a Spy construct, specifically designed on the SpyTag component, with the aim of proving that the Spy protein can be equipped with a specific binding site for metal ions (Figure 2). To this end, we introduced an ATCUN site into the SpyTag peptide. The ATCUN portion (amino terminal copper and nickel binding site) consists of a H_2_N‐Xxx‐Yyy‐His sequence and is known for its ability to bind Cu^2+^ and Ni^2+^ ions. Here we demonstrate that an ST peptide carrying the ATCUN fragment displays high‐affinity binding for Cu^2+^ ions. This affinity is retained in the reconstituted SC/ST protein also when the SpyTag sequence is incorporated at the N‐terminus of a natural unrelated protein such as *E. coli* thioredoxin (Figure 2B). Furthermore, the Cu^2+^ ATCUN metal site presents higher catalytic activity in reactive oxygen species (ROS) production in the reconstituted Spy protein compared to the same site in the SpyTag peptide alone.

This construct therefore represents a solid proof of concept of the possibility to assembly SpyTag components in a lego‐like fashion, providing them the motifs to bind different metal sites. These tunable adducts could then be clicked onto the SpyCatcher to straightforwardly generate novel artificial metalloproteins.

## 2 Results and Discussion

We decided to incorporate a copper(II) ion to assess the feasibility of designing an artificial metalloprotein using the SpyCatcher/SpyTag construct. Copper(II) has the advantage of being spectroscopically active, allowing to collect ligand‐field absorption spectra that provide highly informative details about the first coordination shell. Moreover, following the Irving–Williams series, copper(II) is the bivalent first‐row transition metal that forms complexes with the highest stability.^[^ ^50^, ^51^, ^52^ ^]^

**Figure 3:**
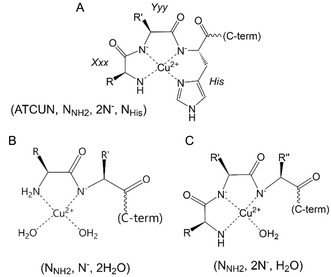
A) Representation of the coordination of copper(II) at the ATCUN (NH_2_‐Xxx‐Yyy‐His) site. B,C) Coordination modes of Cu^2+^ at non‐ATCUN N‐termini of proteins.

We introduced ATCUN H_2_N‐Xxx‐Yyy‐His triads in the SpyTag sequence, in order to create a site that could bind copper(II) with very high affinity. Indeed Cu^2+^ is bound at ATCUN with an equatorial 4N coordination environment (**Figure** **3**A), with formation constants that are remarkably high (*K*_f_ = 10^12^–10^15^ M) and almost independent from the nature of the first two amino acids.^[^ ^53^, ^54^ ^]^ Cu^2+^ binding to the ATCUN site has been extensively studied by optical spectroscopies, and its presence results in a peculiar spectroscopic fingerprint both in terms of UV‐vis absorption (*λ*_max_ ≈ 525 nm) and CD (positive Cotton effect at 490 nm, negative Cotton effect at ≈560–570 nm).

### 2.1 Design of SpyTag Sequences

We first designed two short ATCUN SpyTag peptides, designated **ST‐A1** and **ST‐A2**, and an ATCUN SpyTag peptide located upstream of an *Escherichia* *coli* thioredoxin (EcTrx) domain (**ST‐A3)**. All sequences are reported in Table 1. Both **ST‐A1** and **ST‐A3** bear the same GSH N‐terminus resulting, in the latter construct, from the removal of a 6× His‐tag using a thrombin cleavage site (see below). Conversely, **ST‐A2** features a DAH triad that corresponds to the ATCUN site of human serum albumin, which is considered a paradigmatic ATCUN protein.^[^ ^55^, ^56^ ^]^ Moreover, all three SpyTag peptides bear the VPT triad which kinetically facilitates the formation of the covalent adduct with SC.^[^ ^39^ ^]^ Finally, **ST‐A1** and **ST‐A2** differ by one residue at the C‐terminus: instead of a lysine (K), **ST‐A2** ends with an amidated tyrosine residue. This mutation does not change the total charge of the segment downstream to the consensus sequence. This allowed us to explore the effect of a C‐term SpyTag amidation under conditions in which the total charge at the C‐terminus remains unaltered. The peptides were synthesized by solid‐phase peptide synthesis, purified by RP‐HPLC, and lyophilized (details in Supporting Information).

The **ST‐A3** construct was designed as an *E. coli* thioredoxin fusion derivative bearing an ATCUN SpyTag peptide upstream to the Trx sequence (Table 1). EcTrx is a stable globular protein, known not to bind Cu^2+^ specifically, that we used here for two main purposes. On one hand we aimed to use SDS‐PAGE to follow the reconstitution of the ST/SC adduct, on the other, we aimed to study possible unspecific Cu^2+^ binding equilibria to ATCUN SpyTag fragments when they are fused with unrelated protein domains. The ATCUN triad in **ST‐A3** is GSH, the same as ST‐A1, where the initial GS dyad results from in vitro proteolytic cleavage of the purified recombinant protein. The latter step, which was required to remove the HisTag ((His)_6_) sequence present at the N‐terminus of the expressed protein as a purification handle, was carried out using thrombin (see Table 1). Proteolytic cleavage left the GS dyad at the N‐term which, together with the subsequent His residue, provided the ATCUN site.

Three mutations, namely H6V, W28N, and W31Q, were introduced in the EcTrx‐based, **ST‐A3** construct. The H6V mutation was meant to prevent interaction with Cu^2+^. The other two mutations (W28N and W31Q), instead, were instrumental to have a single fluorescent Trp residue only present on the SpyCatcher component (see below). All three mutation sites were identified by rational design and their effect on the overall protein structure was verified with the use of the AlphaFold2 prediction software.^[^ ^57^ ^]^ In the final sequence, H6V and W31Q are located on the surface of the protein within a β‐strand region and in an external loop, respectively, while the Asn residue at position 28 forms contacts similar to those of Trp28 in the wild‐type protein. The entire sequence of the recombinant protein and the purification procedure are reported in Table 1 and in Supporting Information (Section ‘Protein expression and purification’).

### 2.2 SpyCatcher Design

The Spy construct was redesigned based on the sequence of the second‐generation Spy (SC002). As demonstrated by Howarth and coworkers,^[^ ^37^, ^39^, ^58^ ^]^ the evolution from the first to the third Spy generation led to an improvement of the affinity and of the reaction rate between the SpyTag and the SpyCatcher.^[^ ^37^, ^39^, ^40^, ^58^ ^]^ The crystal structure of SpyCatcher SC002 has not been reported, but a model of its SpyTag adduct was obtained through the AlphaFold2 suite (see Supporting Information). The model indicated that about 25 N‐terminal residues form a nonstructured region with a very low accuracy score. The remaining portion of the polypeptide is successfully predicted and corresponds to the beta‐sandwich domain. This model is in agreement with the X‐ray structure of first‐generation ST/SC, in which the structure of the N‐terminal region of the SC polypeptide is also not defined. The SC002 N‐terminal residues were retained in our design to facilitate SD‐PAGE studies, although not essential for the reaction with SpyTag (PDB 4MLI).

Some amino acid residues were changed in SC in order to favor the Cu^2+^ anchoring points present on ST. In particular, the literature SC002 sequence includes two histidine residues at positions 26 and 62 (see Table 1), which could bind copper(II) with moderate to high affinity. These two residues were mutated into glutamines (H26Q and H62Q) in our design. Specifically, in the case of the first mutation, the Gln substitution was meant to preserve the hydrogen bonding interaction with D65. Importantly, while the residue at position 62 was actually glutamine in first‐generation SC (SC001), H26 is present in all of the updated SC versions. Structural predictions were then performed using the AlphaFold2 software to test the effect of the mutations (H26Q‐H62Q) on the folding of the resulting protein (**SCm**) and its interaction with ST. The structural model of **SCm1/ST‐A1** was perfectly superimposable on the X‐ray structure of SC001/ST001 (Figure S1, Supporting Information) and on the predicted structure of SC002. In fact, the two mutated Gln residues are predicted to keep the same orientation and contacts as those established by the wild‐type His residues.

Two specific proteolytic cleavage sites were introduced downstream to the HisTag (TEV: ENLYFQS and thrombin: LVPRGS) to allow its removal from SC after purification. Following proteolytic digestion of the purified recombinant protein with TEV or thrombin, the amino acid sequences of the resulting SCs, designated as **SC1m** and **SC2m**, respectively, are almost identical, with the exception of the N‐terminal ends (Table 1). The sequences of all recombinant proteins and the procedures utilized for their purification are reported in the Supporting Information (Table S1, Supporting Information, and section “Protein expression and purification”).

### 2.3 Cu^2+^ Binding to ATCUN SpyTag

**Figure 4:**
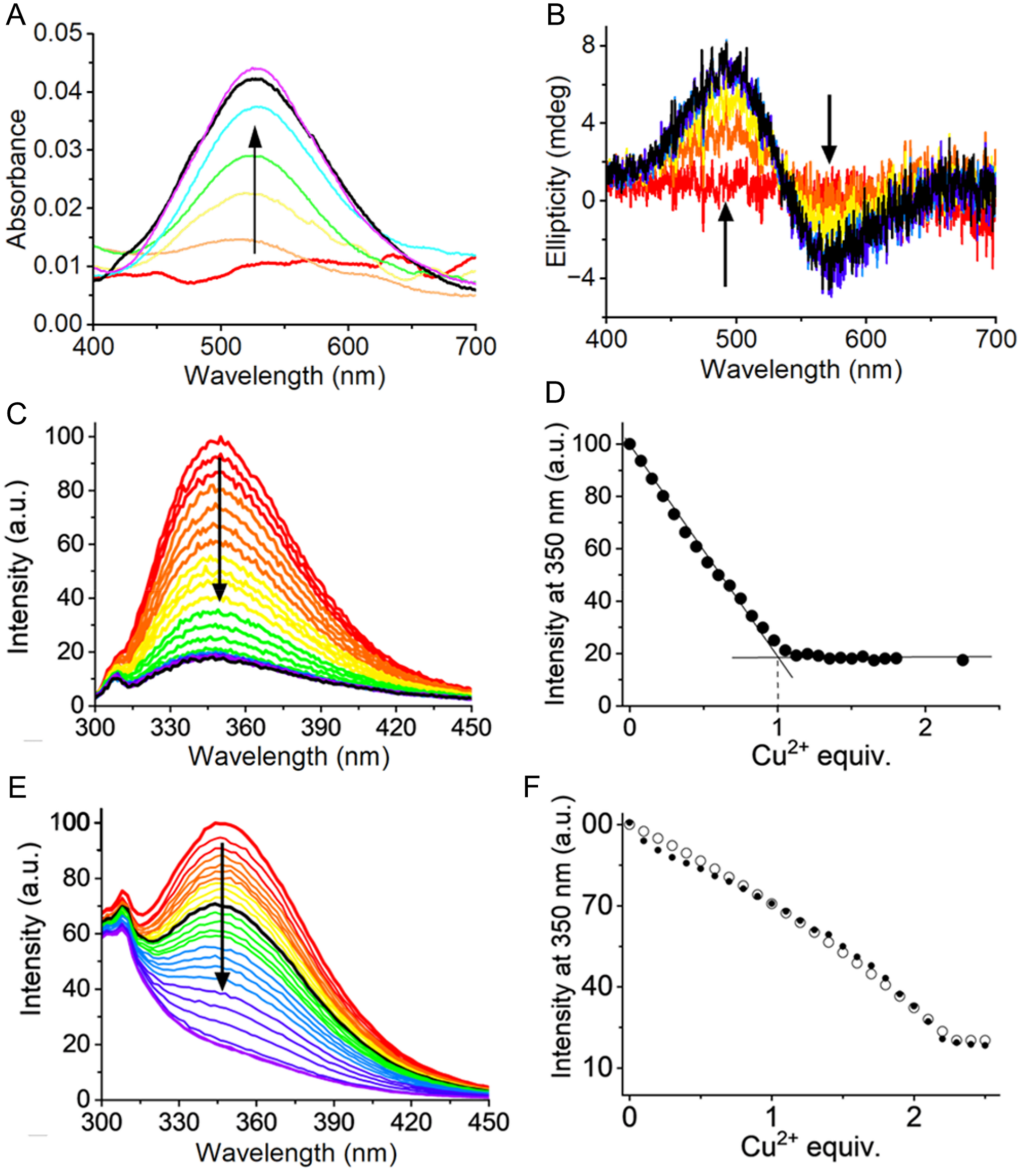
A) Visible absorption spectra for the titration of **ST‐A1** with Cu^2+^ (*C*_ST_ = 400 μM, 20 mM aqueous HEPES buffer, 0.1 M NaCl solution, pH 7.4). Cu^2+^: peptide = 0 (red spectrum) to 1.2 (purple spectrum), with 0.2 eq. additions. B) CD spectra for the titration of **ST‐A1** with Cu^2+^ (same conditions as in (A)). Cu^2+^: peptide = 0 (red spectrum) to 1.25 (purple spectrum), with 0.25 eq. additions. C) Normalized fluorescence emission spectra and D) intensity of emission at 350 nm in function of Cu^2+^ equivalents for the titration of a solution of **AAHAWG‐NH**_**2**_ with Cu^2+^ (*C*_peptide_ = 48 μM, Cu^2+^:peptide = 0–2.3, 25 mM HEPES, pH 7.4). See ref. [59], data available from the authors. E) Normalized fluorescence emission spectra and F) intensity of emission at 350 nm in function of Cu^2+^ equivalents (F) for **ST‐A1** and **AAHAWG‐NH**_**2**_ with up to 2.5 eq. of Cu^2+^ (*C*_ST_ = *C*_AAH_ = 10 μM, 20 mM HEPES, 0.1 M NaCl, pH 7.4). Cu^2+^: peptide = 0 (red spectrum) to 2.5 (purple spectrum), with 0.1 eq. additions. In all plots, the spectra for 1 eq. of Cu^2+^ (vs. SpyTag) are depicted in black.

We started our study by examining the interaction of Cu^2+^ with the Tag peptides using UV‐Vis absorption and CD spectroscopies at physiological pH (20 mM HEPES, 0.1 M NaCl, pH 7.4). Upon addition of 1 Cu^2+^eq., UV‐Vis titration spectra of **ST‐A1**, **ST‐A2**, and **ST‐A3** displayed an absorption peak at 525 nm (**Figure** **4**A, Figure S3A and S6A, Supporting Information). This maximum absorption wavelength is consistent with Cu^2+^ bound in the equatorial (4N) coordination at the ATCUN site. We analogously collected CD spectra of the same UV‐Vis samples to confirm our design. Indeed, the distinctive CD spectrum profile of a Cu^2+^/ATCUN complex appeared upon the addition of 1 eq. of Cu^2+^ for all of the Tag peptides, with a positive band at 490 nm and a negative one at ≈560–580 nm (Figure 4B, Figure S3B and S6B, Supporting Information).^[^ ^59^ ^]^ These spectroscopic features indicate that Cu^2+^ binds to **ST‐A1**, **ST‐A2**, and **ST‐A3** at the ATCUN site, as predicted by our design.

The Cu^2+^ binding affinity of ATCUN sites was determined by spectrofluorimetric titrations. We decided to carry out competition fluorimetric titrations using the short **AAHAWG‐NH**_**2**_ ATCUN peptide as a fluorescent competing ligand, which forms a high‐affinity complex with copper(II) (log *K*_f_ = 13.5, see Supporting Information).^[^ ^59^ ^]^ The presence of a Trp residue in this peptide results in an emission at 350 nm which is completely quenched upon the addition of 1 eq. of copper(II) (Figure 4C,D). In this way, we circumvented the absence of a Trp residue in the SpyTag peptides, still observing an intense emission and a significative quenching upon Cu^2+^ binding. A representation of the spectral dataset for the **ST‐A1**, **ST‐A2** titrations is reported in Figure 4E and Figure S5, Supporting Information. A detailed description of the experimental setup is provided in Supporting Information, along with additional spectral dataset representations for **ST‐A3** (Figure S8, Supporting Information).

The steep decrease in intensity upon the addition of Cu^2+^ to the competing **AAHAWG‐NH**_**2**_ ATCUN peptide was observed, with a nearly complete luminescence quenching upon the addition of 1 metal ion equivalent (Figure 4C,D). Conversely, the presence of the ATCUN SpyTag peptides resulted in strong competition for Cu^2+^ binding, and therefore, only a limited fluorescence quenching was observed upon the addition of 1 metal ion equivalent (Figure 4E, Figure S5A and S8A, Supporting Information). Indeed, complete emission quenching was observed upon the addition of 2 Cu^2+^ equivalents (Figure S8B, Supporting Information, Figure 4F and Figure S5B, Supporting Information). This result is consistent with the saturation of the ATCUN sites of **AAHAWG‐NH**_**2**_ and those of the SpyTag peptides. Data analysis with the HypSpec2014 software yielded log *K*_f_ for the three ATCUN **ST‐A1**, **ST‐A2**, and **ST‐A3** peptides ranging from 13.7 to 13.9 (**Table** **2**). These log *K*_f_ values are fully consistent with those expected for the binding of Cu^2+^ at an ATCUN site.

**Table 2:** Logarithms of conditional Cu^2+^ complex formation constants (log *K*_f_) with SpyTag and SpyCatcher (20 mM aqueous HEPES buffer, 0.1 M NaCl solution, pH 7.4). Standard deviations are given in parentheses.

Ligand	Log *K*_f_	Coordination mode
**ST‐A1**	13.7(1)	ATCUN GSH
**ST‐A2**	13.9(1)	ATCUN DAH
**ST‐A3**	13.8(1)	ATCUN GSH
**SC1m**	5.1(1)	N‐term (NH_2_, N^−^, 2H_2_O)
**SC2m**	5.6(1)	N‐term (NH_2_, 2N^−^, H_2_O)

Finally, we attempted a direct spectrofluorimetric titration of Cu^2+^ binding to **ST‐A3** by measuring the emission of the Tyr residues at 305 nm. Interestingly, the addition of up to 11 Cu^2+^ eq. to **ST‐A3** resulted in a relatively small quenching of Tyr emission (Figure S7A, Supporting Information). Most importantly the titration did not reach a clear titration endpoint upon addition of 1 copper(II) eq. (Figure S7B, Supporting Information). This result could be due to nonspecific binding of the metal ions to **ST‐A3** with binding to the ATCUN site not clearly evidenced using this approach because the tyrosine resides are distant from ATCUN unit. Perhaps expectedly, this result supports the choice of short peptides such as **ST‐A1** or **ST‐A2** to design new artificial constructs. The strategy allows one to maximize the role of high‐affinity metal binding sites, while reducing the impact of undesired nonspecific metal binding.

### 2.4 Cu^2+^ Binding to the SpyCatcher

**Figure 5:**
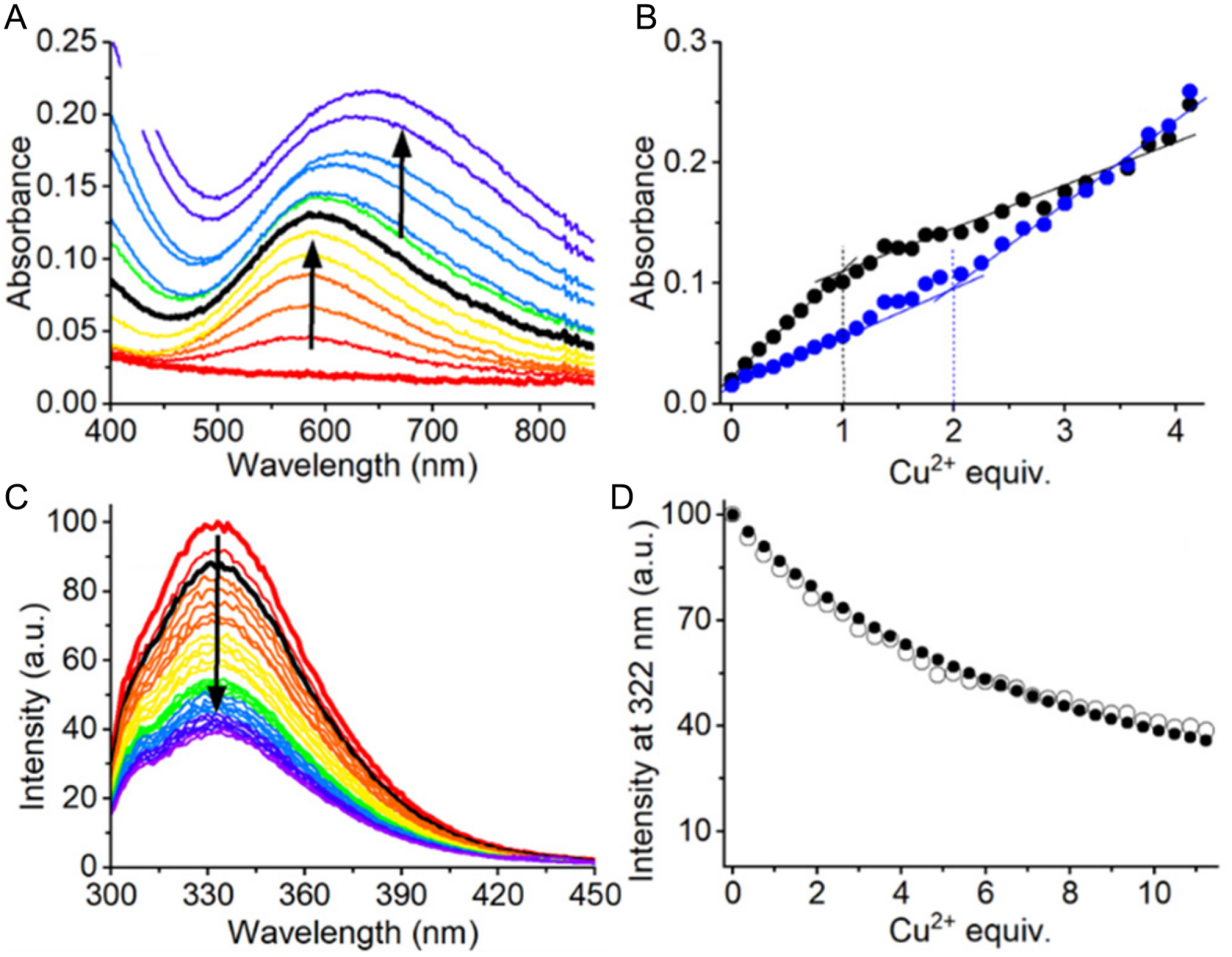
A) Visible absorption spectra for the titration of **SC2m** with Cu^2+^ (*C*_SC_ = 948 μM, 20 mM aqueous HEPES buffer, 0.1 M NaCl solution, pH 7.4). Cu^2+^: protein = 0 (red spectrum) to 4.125 (purple spectrum), with 0.125 eq. additions. B) Absorption values for the isible titration of **SC2m** with Cu^2+^ at 580 nm (black) and 700 nm (blue) as a function of Cu^2+^ equivalents. C) Fluorescence emission spectra and D) intensity of emission at 322 nm in function of Cu^2+^ equivalents for the titration of a solution of **SC2m** with up to 12 eq. of Cu^2+^ (*C*_SC_ = 10.0 μM, 20 mM HEPES, 0.1 M NaCl, pH 7.4). Cu^2+^: peptide = 0 (red spectrum) to 12.0 (purple spectrum) with 0.35 eq. additions. In all plots, the spectra for 1 eq. of Cu^2+^ are depicted in black.

The Cu^2+^ binding to **SC1m** and **SC2m** was analyzed by UV‐Vis absorption and CD spectroscopies. We first titrated **SC1m** and **SC2m** with up to 1 eq. of Cu^2+^, observing an increase of the bands at ≈650 and 580 nm, respectively (**Figure** **5**A and Figure S9A, Supporting Information). These bands can be ascribed to the binding of Cu^2+^ to the N‐terminus of the SpyCatcher constructs, with formation of a (N_NH2_, N^−^, 2H_2_O) or a (N_NH2_, 2N^−^, H_2_O) copper(II) coordination in the case of **SC1m** and **SC2m**, respectively (Figure 3B,C).^[^ ^60^ ^]^ Additionally, in the case of **SC2m** the addition of more than 1 Cu^2+^ equivalent resulted in a *red* *shift* together with broadening of its visible absorption band, as the consequence of the onset of a second absorption band at ≈700 nm, which becomes visible in the spectra collected with an excess Cu^2+^ (Figure 5A).

Plotting molar absorbance at 580 and 700 nm as a function of the Cu^2+^ equivalents added to **SC2m** revealed inflection points at 1 and 2 eq. of Cu^2+^, respectively (Figure 5B). In parallel, a negative Cotton signal at 590 nm appeared in the CD spectrum of **SC2m** for the addition of a second Cu^2+^eq. (Figure S11, Supporting Information). Addition of Cu^2+^ beyond 1 eq. to **SC1m** resulted in similar behavior, with appearance of a negative band at ≈490 nm in the CD spectrum (Figure S9B, Supporting Information). Overall, these observations suggest the binding of Cu^2+^ at the N‐terminus in both **SC1m** and **SC2m**, possibly accompanied by nonspecific binding to carboxylate‐rich regions in the presence of excess metal ions (Figure S19, Supporting Information).

The affinity of the **SC1m** and **SC2m** proteins for Cu^2+^ at the N‐terminus was determined by direct fluorescence titration, following the quenching of the emission of the Trp residue at 350 nm. A limited but significant (≈15% for **SC1m** and 20% for **SC2m**) fluorescence quenching was observed upon the addition of 2 eq. of Cu^2+^. The effect increases to 50% and 60% quenching for **SC1m** and **SC2m**, respectively, upon the addition of a larger excess of copper(II) (≈11 eq., Figure 5C,D, Figure S10, Supporting Information). Analysis of quenching data provided a log *K*_f_ of 5.1(1) for **SC1m** and 5.6(1) for **SC2m** (Table 2). Both are consistent with that of Cu^2+^ to pentaglycine (log *K*_f_ = 4.0 in MOPS buffer, log *K*_f_ = 6.17 in 0.1 M KCl calculated from potentiometric data).^[^ ^60^, ^61^ ^]^ Importantly, the fluorescence dataset did not allow us to determine the binding constant for the second Cu^2+^ ion, even though analysis of model equilibria with the Hyss software suggests a stepwise log *K*_f_ for the binding of the second Cu^2+^ lower than 3.^[^ ^62^ ^]^

Overall, the above spectroscopic data indicates that the primary copper binding site is the N‐terminus for **SC1m** and **SC2m**, as predicted by our design. The affinity for copper(II) of the N‐termini of both SC proteins turned out to be ≈9–10 orders of magnitude lower than that of the ATCUN site of the SpyTag peptides. This difference in affinity strongly supports that selective and sequential binding of Cu^2+^ at these two sites may occur when the ST/SC adduct is formed.

### 2.5 Formation of the SpyCatcher/SpyTag Complex

The covalent binding between **ST‐A1** and **ST‐A2** and **SC1m** was studied by LC‐MS, comparing chromatograms and *m/z* ratios of the ST/SC samples with those of individual components (Figure S20 and S21, Supporting Information). Solutions of **ST‐A1**, **ST‐A2**, and **SC1m** in 20 mM HEPES, pH 7.4 (25 μM each), were incubated at room temperature. After 5 min, the mixtures were injected into the HPLC and analyzed by MS. The diagnostic MS peak corresponding to the SpyTag could no longer be detected in the chromatograms 5 min upon their incubation of either **ST‐A1** or **ST‐A2** to **SC1m** solution. Moreover, ion patterns consistent with their corresponding ST/SC complexes were observed (Figure S20B and S21B, Supporting Information). These results indicated that ST/SC complex formation was practically completed in less than 5 min.

**Figure 6:**
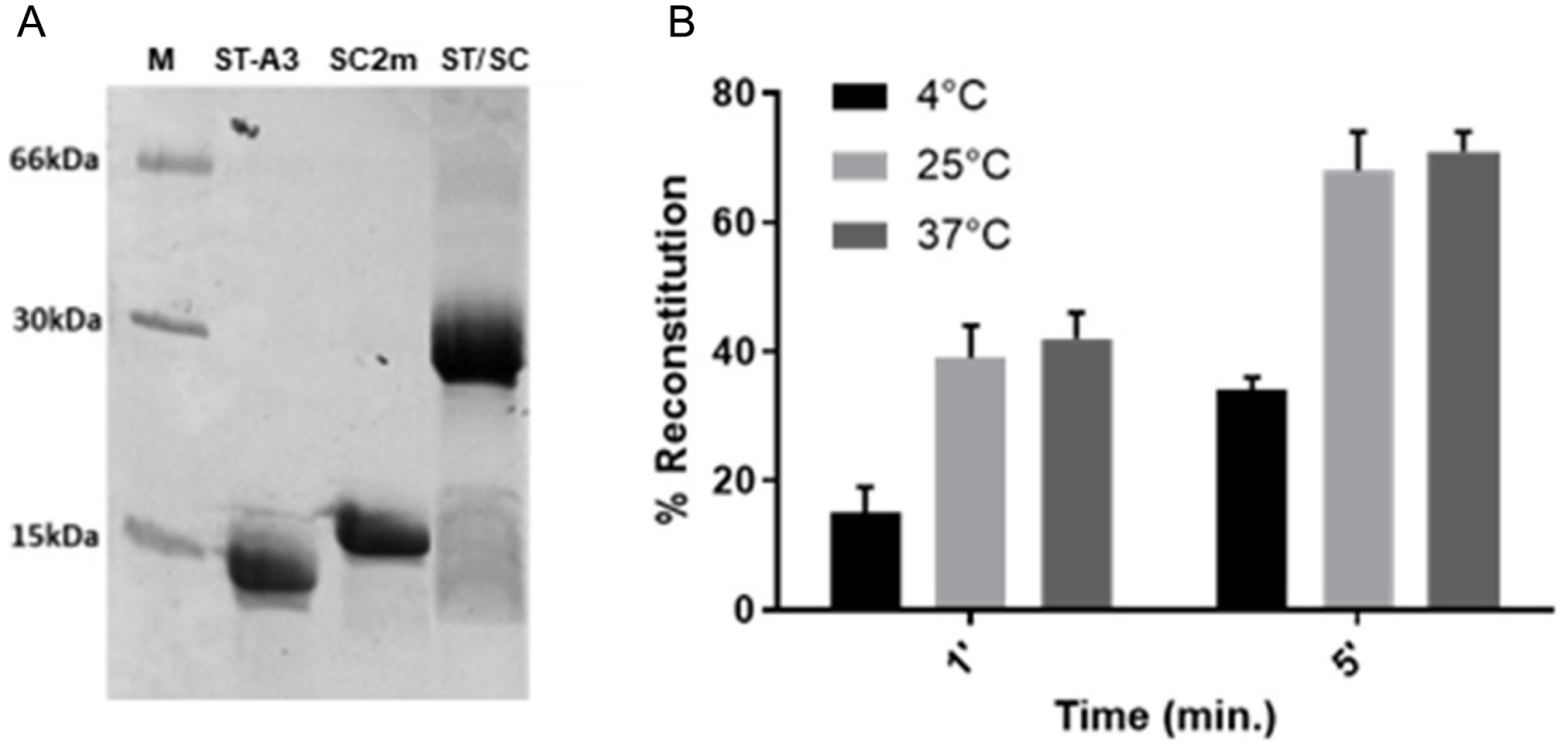
A) SDS‐Page analysis of **SC2m** and **ST‐A3** (10 μM each in Tris/HCl buffer 25 mM, 0.15 M NaCl, pH 7.5) incubated for 15 mins at room temperature. B) Temperature dependence of the reaction of **SC2m** and **ST‐A3** at 10 μM each, analyzed by SDS‐PAGE. Error bars represent the standard deviation of the mean calculated from three independent replicates for each reconstitution experiment.

Covalent binding between SpyTag and SpyCatcher was also analyzed by SDS‐PAGE using **SC2m** and **ST‐A3** (**Figure** **6**). When equimolar amounts of **SC2m** and **ST‐A3** (10 μM each) were incubated for 15 min at room temperature in Tris/HCl buffer (25 mM, pH 7.5), 0.15 M NaCl, complete conversion of the **ST‐A3** peptide into a covalent ST/SC complex of the expected size was observed (Figure 6A). The complex formation between **SC2m** and **ST‐A3** was also studied as a function of temperature within a 1–5 min time window (Figure 6B). Nearly 40% of product formation was already formed upon 1 min at 25 °C. The yield rose to 70% after 5 min. Similar values were observed at 37 °C. Expectedly, the process was slower at 4 °C and the incubation of SC with ST yielded only 17% and 35% of ST/SC product after 1 and 5 min, respectively.

Same complex reconstitution experiments carried out in the presence of Cu^2+^ and analyzed with the use of LC‐MS and SDS‐PAGE demonstrated that the presence of the metal ion in the reaction mixture does not affect ST/SC adduct formation (Figure S22 and S23, Supporting Information).

### 2.6 Cu^2+^ Binding to SpyCatcher/SpyTag Complex

The design of a selective Cu^2+^ binding site on a protein is highly challenging because of the competition of the N‐terminus of the protein which may act as an alternative (*i.e.* secondary) binding site. To obtain selective binding of the Cu^2+^ metal cofactor to SpyTag in the presence of SpyCatcher, we thus incorporated ATCUN peptide fragments into the former component. Based on this design, we predicted the selective binding of Cu^2+^ to the ATCUN site of the reconstituted ST/SC adduct when the metal ion is present in an equimolar amount with respect to the protein.

**Figure 7:**
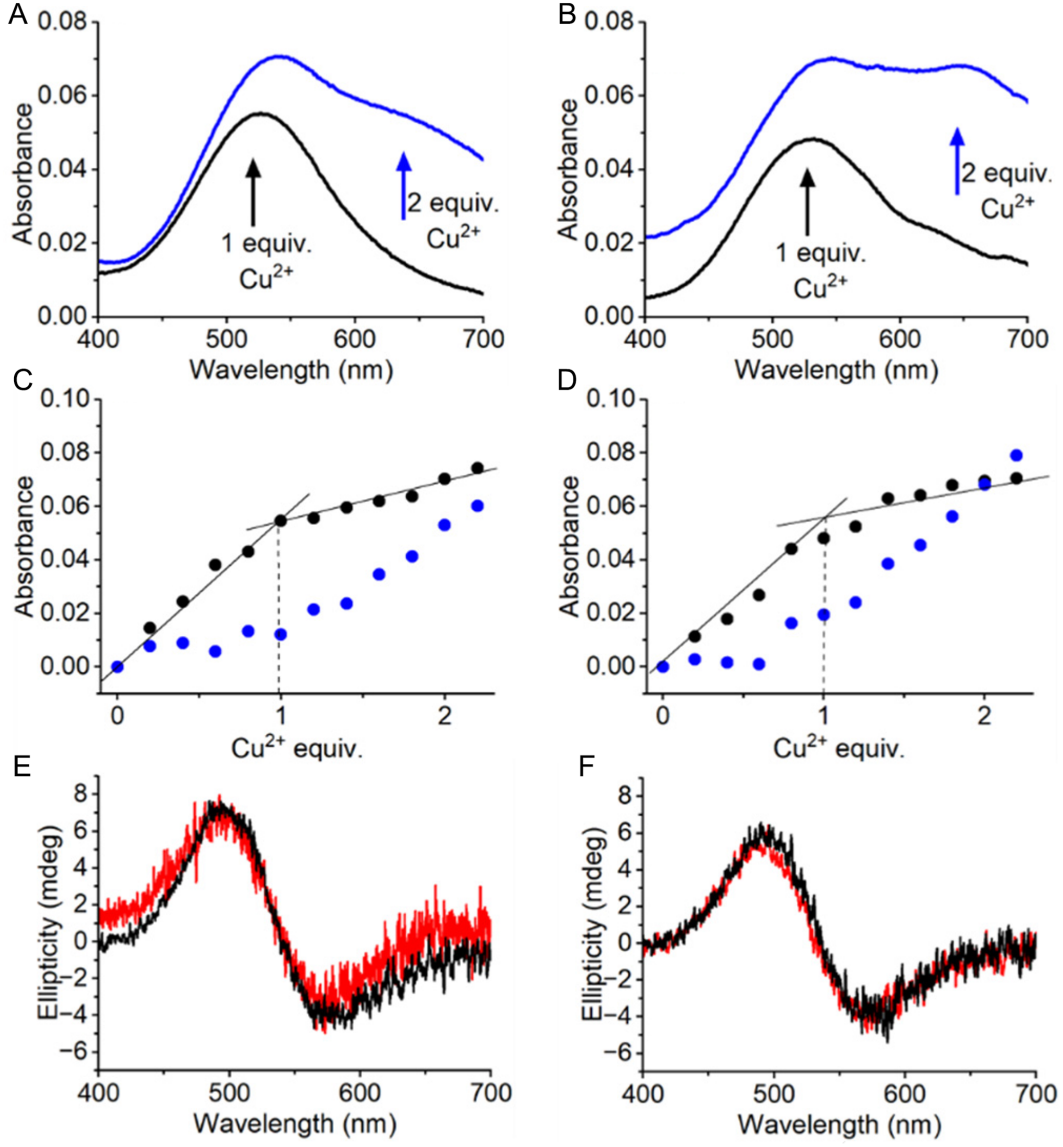
Visible absorption spectra of A) **ST‐A1/SC1m** and B) **ST‐A2/SC1m** added with 1 equivalent (black) or 2 equivalents (blue) of Cu^2+^. (*C*_SC2m_ = *C*_ST‐A_ = 400 μM, 20 mM aqueous HEPES buffer, 0.1 M NaCl solution, pH 7.4). Absorption values for the visible titration of C) **ST‐A1/SC1m** or D) **ST‐A2/SC1m** with Cu^2+^ at 535 nm (black) and 650 nm (blue) as a function of Cu^2+^ equivalents. CD spectra of E) **ST‐A1/SC1m** (black) and **ST‐A1** (red) both with 1 eq. of Cu^2+^ and F) **ST‐A2/SC1m** (black) and **ST‐A2** (red) both with 1 eq. of Cu^2+^ (*C* = 400 μM, 20 mM aqueous HEPES buffer, 0.1 M NaCl solution, pH 7.4).

Copper(II) binding to the ST/SC adducts was first investigated by UV‐Vis titrations. **SC1m** was incubated for 15 min at room temperature with **ST‐A1** or **ST‐A2** and then titrated with copper(II). The addition of up to 1 equivalent of the metal ion to either **ST‐A1/SC1m** or **ST‐A2/SC1m** resulted in a progressive increase of a band at 525 nm (**Figure** **7**A,B). These results are fully consistent with those observed for the binding of copper(II) at the ATCUN site of the simpler ST (Figure 4 and Figure S3, Supporting Information).

The addition of an amount of copper(II) higher than 1 eq. resulted in the appearance of a second absorption signal in the form of a *red* *shifted* shoulder of the main Cu^2+^/ATCUN band for both **ST‐A1/SC1m** and **ST‐A2/SC1m**, (Figure 7A,B). Differential spectra were derived by subtraction of the spectrum recorded in the presence of 1 Cu^2+^ eq. from the uncorrected spectral dataset (Figure S12C and S12F, Supporting Information) showed that the absorption band observed upon addition of the second Cu^2+^eq. is centered around 650 nm, which is fully consistent with the absorption peak observed with **SC1m** alone (Figure S9A, Supporting Information). This behavior indicates that the binding of the second Cu^2+^ ion to **ST‐A1/SC1m** and **ST‐A2/SC1m** takes place at the N‐terminus of the SpyCatcher component, in agreement with our initial design.

The binding of the first equivalent of copper(II) to the ATCUN SpyTag peptides of the ST/SC complexes was further confirmed by circular dichroism. Indeed, CD spectra recorded after the addition of 1 Cu^2+^eq. to the **ST‐A1/SC1m** or the **ST‐A2/SC1m** showed a positive band at 490 nm and a negative band at ≈560–570 nm. Both bands are superimposable on those observed with the two SpyTag peptides alone (Figure 7E,F, comparable with Figure 4B and Figure S3B, Supporting Information).

**Figure 8:**
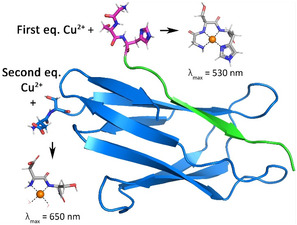
Representation of the two Cu^2+^ binding sites in the ATCUN SpyTag/SpyCatcher adducts. The coordination modes at the ATCUN and SC N‐Term sites are represented, along with the observed wavelength of ligand field absorption maxima.

Overall, the spectroscopic data obtained for the binding of Cu^2+^ to the SpyTag/SpyCatcher adducts support a model fully consistent with our design: the primary binding of Cu^2+^ to the ATCUN site of the SpyTag component, followed by metal binding to the N‐term of the SpyCatcher (**Figure** **8**). With this information in our hands, we have determined the affinity of the reconstituted **ST‐A1/SC1m** or the **ST‐A2/SC1m** proteins for Cu^2+^ by spectrofluorimetric competition titration, using the same approach used for the SpyTag peptides (**AAHAWG‐NH**_**2**_ peptide as the competitor, Figure S13A and S14A, Supporting Information). The *K*_f_ of binding of Cu^2+^ to the ATCUN sites in **ST‐A1/SC1m** or the **ST‐A2/SC1m** resulted 14.5(1) and 13.7(2), respectively. Using these constants, we have treated the spectra dataset of Figure S15 and S16, Supporting Information by including a 2:1 Cu^2+^/protein adduct in the model, along with the 1:1. The refined *K*_f_ values for the binding of the second copper(II) at the N‐terminus of SC resulted in 3.3(1) and 3.4(1) for **ST‐A1/SC1m** or the **ST‐A2/SC1m**. These data show that we reached our objective of designing a modularly assembled protein bearing an ATCUN site that is selectively occupied by Cu^2+^ in the presence of the protein N‐terminus.

Finally, UV‐vis spectroscopic analysis of the **ST‐A3/SC2m** adduct provided results consistent with those obtained for **ST‐A1/SC1m** and **ST‐A2/SC1m**. These results are described in detail in the Supporting Information.

### 2.7 Catalytic ROS Production

Although known since decades, only in recent years Cu^2+^/ATCUN systems gained attention as potential catalysts of chemical reaction of biological importance such as the production of ROS.^[^ ^63^, ^64^, ^65^ ^]^ To assess the capacity of our Cu^2+^ ATCUN systems to operate catalytic activity in ROS production, we have used 2,7‐dichlorofluorescein diacetate (DCHF‐DA) as a probe. DCHF‐DA is a profluorescent dye which reacts with hydroxyl radicals to form 2,7‐dichlorofluorescein in solution.^[^ ^66^ ^]^ The assay was performed in the presence of hydrogen peroxide and ascorbate which are commonly used to promote hydroxyl radical formation and catalytic turnover in copper‐redox reactions.^[^ ^52^, ^55^, ^64^ ^]^

**Figure 9:**
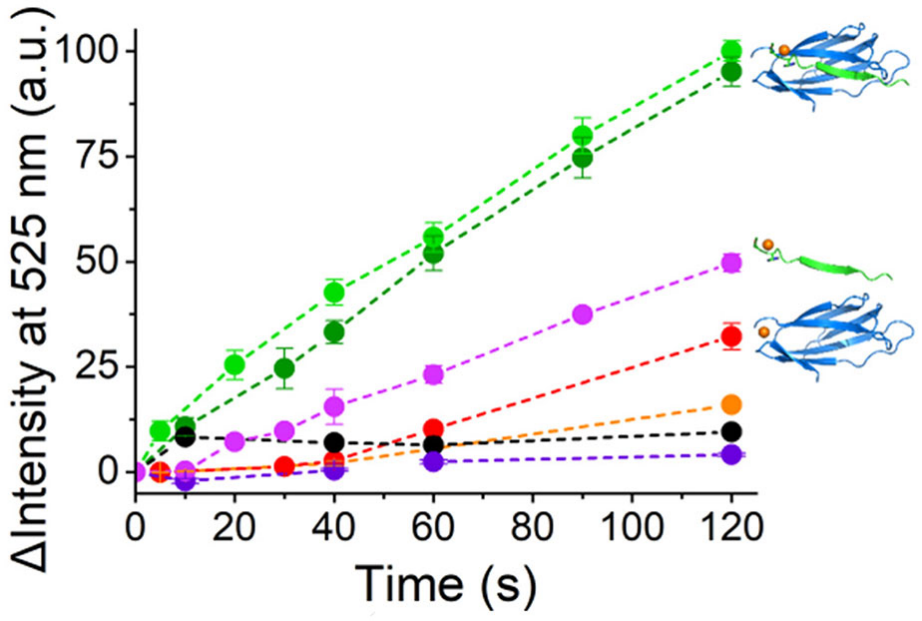
Increase in intensity of fluorescence emission at 525 nm due to ROS production in the presence of DCHF‐DA. Black: Cu^2+^; orange: **SC1m**; red: **SC1m**:Cu^2+^ 1:1; magenta: **ST‐A1**:Cu^2+^ 1:1; dark green: **ST‐A1**:Cu^2+^ 1:1 added with 1 eq. of **SC1m**; pale green: **ST‐A1:SC1m** 1:1 added with Cu^2^ Error bars result from averaging the values from three independent replicates. (*C*_peptide_ = 10 μM, *C*_SC2m_ = 10 μM, *C*_DCHF‐DA_ = 100 μM, 25 mM HEPES, 0.1 M NaCl, pH 7.4).

Data in **Figure** **9** show the increase of the emission of the fluorescent probe due to hydroxyl radical production when 1 eq. of Cu^2+^ is added to **ST‐A1**/**SC1m**, **ST‐A1**, or **SC1m** (green, magenta, and red traces, respectively). As for the green traces, they refer to Cu^2+^ added to **ST‐A1** before or after the addition of **SC1m** (dark and light green, respectively). It is evident that the Cu^2+^ ATCUN site in the reconstituted **ST‐A1**/**SC1m** protein is the fastest in promoting the production of hydroxyl radicals under the examined conditions. Compared to the reconstituted protein, Cu^2+^ bound at **ST‐A1** or **SC1m** are slower by a factor of ≈2 and 3, respectively (magenta and red traces). Although these results are overall in agreement with previous characterizations reported in literature for other ATCUN motifs,^[^ ^62^, ^67^, ^68^, ^69^ ^]^ data in Figure 9 show that the presence of the **SC1m** protein makes the Cu^2+^/ATCUN site of **ST‐A1** more efficient in ROS production compared to the metallopeptide alone. To explain this difference in behavior, we put forward the hypothesis that the Cu^2+^/ATCUN interacts with some sites on the surface of the reconstituted **ST‐A1**/**SC1m** protein. Possibly, a distortion from the square planar coordination of Cu^2+^ at ATCUN which favors the Cu^+^/Cu^2+^ redox cycle or a modulation of the redox potential as a result of second‐shell interactions may occur. This in turn results in the observed higher catalytic activity. Finally, it is interesting that the increase of intensity for Cu^2+^/**ST‐A1**/**SC1m** is in a linear regime over 120 min, suggesting that the metalloprotein is stable over this time frame.

Under the conditions used, all control reactions but Cu^2+^ bound to **SC1m** resulted in negligible ROS production activities (Figure 9 and Figure S18, Supporting Information). Although interesting, we did not investigate this system further in detail since the Cu^2+^/**ST‐A1**/**SC1m** metalloprotein was studied in the presence of 1 eq. of Cu^2+^, which invariably presents the metal ion coordinated to ATCUN and not to the N‐terminus of **SC1m**.

Overall, our results demonstrate that the Spy technology can be successfully exploited to easily assemble metalloproteins in a well‐designed, lego‐like fashion. Most importantly, using this approach, we have provided a metal site on a peptide with a catalytic behavior not possessed by the simple metallopeptide, opening the possibility of designing and straightforwardly assembling similarly complex metal‐containing architectures.

## 3 Conclusion

Although the use of Spy as tagging technology is well established, our construct is the first artificial metalloprotein bearing designed and catalytically active metal‐binding sites associated with a SpyTag/SpyCatcher assembly. Our design introduced ATCUN as a coordination site for the selective binding of 1 eq. of Cu^2+^, which presents a copper(II) binding affinity that is at least 9 orders of magnitude higher than that of other potential metal‐binding sites present on the final construct, including SpyCatcher N‐terminus. This construct proved to be catalytically active in the production of ROS from hydrogen peroxide and ascorbate: possibly most importantly, the Cu^2+^/ATCUN site exhibited a significant catalytic activity only when mounted on the protein construct.

Our results open a window on the de novo design of metalloproteins and metalloenzymes bearing one or more artificial metal‐binding sites, including completely artificial ones. Possibly more important, this approach leverages the specificity of the interaction of the components in this tagging system to introduce entirely artificial metal catalytic sites onto a protein construct lacking such functionalities. Indeed, at the basis of our design strategy, there is a shift in the complexity inherent to the design of metal‐binding sites from a protein to a peptide. Since peptide synthesis is in general quite straightforward, the insertion of one or more catalytic, photo‐, or electrocatalytic metal sites can be performed on SpyTag. Following the introduction of the metal center into the engineered peptide, the resulting functionalized SpyTag can be clicked onto the SpyCatcher, leading to a protein construct derivative bearing one or more grafted metal‐binding sites. Possibly most importantly, SpyCatcher, as a small protein, can be fused to many other protein systems opening to the possibility to exploit the modularity of the system using SpyTag components on which desired artificial functional groups are mounted. We thus anticipate broad future applications of the present concept.

## Conflict of Interest

The authors declare no conflict of interest.

## Author Contributions

**Silvia Gentili**: data curation (equal); investigation (equal); writing—original draft (equal). **Francesca Miglioli**: data curation (equal); investigation (equal); writing—original draft (equal). **Valentina Borghesani**: data curation (equal); investigation (equal); supervision (equal); writing—original draft (equal); writing—review and editing (equal). **Gloria Spagnoli**: investigation (equal); writing—original draft (equal). **Denise Bellotti**: investigation (equal); writing—original draft (equal). **Davide Cavazzini**: data curation (equal); investigation (equal); writing—original draft (equal). **Remo Guerrini**: investigation (equal); supervision (equal). **Maurizio Remelli**: investigation (equal); writing—original draft (equal). **Giovanni Maestri**: funding acquisition (lead); project administration (equal); writing—original draft (equal). **Simone Ottonello**: conceptualization (equal); writing—original draft (equal). **Angelo Bolchi** conceptualization (equal); investigation (equal); methodology (equal); supervision (equal); writing—original draft (equal). **Matteo Tegoni**: conceptualization (lead); funding acquisition (equal); methodology (lead); project administration (equal); supervision (equal); writing—original draft (equal); writing—review and editing (equal). **Silvia Gentili** and **Francesca Miglioli** contributed equally to this work.

## Supporting information

Supplementary Material

## Data Availability

The data that support the findings of this study are available from the corresponding author upon reasonable request.
