# Supplementary material for: Exploiting SpyTag/SpyCatcher Technology to Design New Artificial Catalytic Copper Proteins
Source: Chembiochem. 2025 May 21;26(14):e202500208. doi: 10.1002/cbic.202500208 (PMC12278347; doi:10.1002/cbic.202500208)
Supplement: Supplementary file 1 — Supplementary Material [file CBIC-26-e202500208-s001.pdf]

# Exploiting The SpyTag/SpyCatcher Technology To Design New Artificial Catalytic Copper Proteins

Silvia Gentili,§ [a] Francesca Miglioli,§ [a] Valentina Borghesani, [a] Gloria Spagnoli,[a] Denise Bellotti,[b] Davide Cavazzini,[a] Remo Guerrini,[b] Maurizio Remelli,[b] Giovanni Maestri,[a] Simone Ottonello,[a] Angelo Bolchi [a] and Matteo Tegoni* [a]

**Table of contents:**

1. MATERIALS ........ 2

2. EXPERIMENTAL PROCEDURES ........ 2

2.1. Peptide synthesis ........ 2

2.2. Protein expression and purification ........ 2

2.3. SpyCatcher/SpyTag reconstitution analysis and quantitation ........ 3

2.4. ESI-MS ........ 3

2.5. Spectroscopic studies ........ 4

3. Supporting Information Figures ........ 9

## 1. MATERIALS

**ST-A1** was purchased by GenScript. The artificial genes coding for the two recombinant proteins SCm and **ST-A3**, designed using the E. *coli* codon-usage, were synthesised by GenScript (Table S1) and cloned into the pET28b vector at the restriction sites NcoI and HindIII. **SC1m/SC2m** and **ST-A3** polipeptides were obtained after the protein expression and purification procedure. All other chemicals were purchased from Merck and used without further purification.

## 2. EXPERIMENTAL PROCEDURES

### 2.1. Peptide synthesis

**AAHAWG-NH$_2$** and **ST-A2** were synthesized according to published methods[71] using Fmoc/*t*-butyl chemistry with a Syro XP multiple peptide synthesizer (MultiSynTech GmbH, Written Germany). Rink amide resin MBHA was used as a solid support for the synthesis of the peptides. Fmoc-amino acids (4-fold excess) were sequentially coupled to the growing peptide chain using DPEA/HATU (N,N-diisopropylethylamine/ O-(7-azabenzotriazol-1-yl)-N,N,N',N'-tetramethyluronium hexafluorophos-phate) (aa:HATU:DPEA = 1:1:2) as activating mixture for 1 h at room temperature. The activating mixture should be carefully handle since HATU, as all the uranium based coupling agents, could induce airborne allergic skin sensitization or induce anaphylaxis.[72,73] Cycles of deprotection of Fmoc (40% piperidine/N,N-dimethylformamide) and coupling with the subsequent amino acids were repeated until the desired peptide-bound resin was completed. The protected peptide-resin was treated with trifluoroacetic acid (TFA)/$H_2O$/triisopropylsilane 90:5:5; v/v; 10 mL per 0.2 g of resin for 1.5 h at room temperature.[74] After filtration of the resin, the solvent was concentrated *in vacuo* and the resin triturated with ethyl ether. Crude peptides were purified by preparative reversed-phase HPLC using a Water Delta Prep 3000 system with a Jupiter column C18(250 × 30 mm, 300 A, 15 µm spherical particle size). The column was perfused at a flow rate of 20 ml/min with a mobile phase containing solvent A (water in 0.1% TFA), and a linear gradient from 0 to 90% of solvent B (60% v/v, acetonitrile in 0.1% TFA) over 35 min for the elution of peptides. Analytical HPLC analyses were performed on Beckman 116 liquid chromatograph equipped with a Beckman 166 diode array detector. Analytical purity of the peptides was assessed using a Zorbax C18 column (4.6 ×150 mm, 3 µm particle size) with the above solvent system (solvents A and B) programmed at a flow rate of 0.7 ml min$^{-1}$ using a linear gradient from 0% to 100% B over 30 min. All analogues showed ≥ 95% purity when monitored at 220 nm. Molecular weight of final compounds was determined by a mass spectrometer ESI Micromass ZMD-2000.

### 2.2. **Protein expression and purification**

SpyCatcher proteins (**SC1m** and **SC2m**) and **ST-A3** were expressed in *E. coli* BL21(DE3) codon plus at 20 °C O/N in LB medium, adding 1 mM IPTG at $OD_{600}$: 0.6. The **SCm** present in the soluble fraction of the bacterial lysate was enriched by a his-tag affinity chromatographic step (FF HiTrap

crude, Cytiva). His-tag was removed by TEV or thrombin digestion (respectively for **SC1m** and **SC2m**), and the SpyCatcher proteins isolated in a pure form with an anion exchange column refinement step (MonoQ, Cytiva). The **ST-A3** protein was isolated from the *E. coli* insoluble fraction. The protein was dissolved in 6 M urea and eluted from a His-tag affinity column after applying an in-column renaturation protocol. After his-tag thrombin cleavage, the **ST-A3** protein was purified to homogeneity by anion exchange chromatography. The protein purity was assessed by SDS-PAGE. The correct size of the isolated proteins was verified by electrospray ionisation mass spectrometry analysis (ESI-MS).

### 2.3. SpyCatcher/SpyTag reconstitution analysis and quantitation

**ST-A1/SC1m and ST-A2/SC1m reconstitution.** The reconstitution experiments between the **SC1m** and **ST-A1** and **ST-A2** were conducted by incubating aqueous solutions of the two components (**SC1m**:**ST** peptides =1:1, $C_{protein}$ = 25 µM) for 5 minutes in 25 mM Hepes buffer and 0.1 M NaCl at pH 7.4. After 5 minutes, 6 µL of the solutions were injected and analysed via LC-MS.

For the experiment in presence of copper(II), solution of **ST-A1**, **ST-A2** and **SC1m** 50 µM (in 25 mM Hepes buffer and 0.1 M NaCl at pH 7.4) were pre-incubated for 1 hour with 50 µM of $CuCl_2$. Then, samples were prepared adding equal amounts of ST and SC2m solution (**SC1m**:**ST** peptides =1:1, $C_{protein}$ = 25 µM), incubated for 5 minutes and analysed as previously descripted.

**ST-A3/SC2m reconstitution.** The reconstitution experiments between the **SC2m** and **ST-A3** proteins were carried out by incubating aqueous solutions of the two proteins at the specified concentration and incubation time in 25 mM Tris/HCl buffer and 0.15 M NaCl at pH 7.5. The reaction was stopped by the addition of SB4X loading dye solution, followed by heating the samples for 10 min at 95 °C. SDS-PAGE analysis of the resulting conjugated and unconjugated proteins was performed in a 15% polyacrylamide gel. Protein complex formation was quantified on SDS PAGE gel after coomassie blue band staining with a ChemiDoc MP Imaging System (Bio-Rad) and the Image-Lab software. The percentage of isopeptide formation at different times and temperatures was calculated by the ratio of the band intensity, using a completed reaction as a reference. In experiments aimed at elucidating the role of copper(II) on recombination, an equimolar amount of $CuSO_4$ was added to the proteins prior to the addition of **ST-A3**.

### 2.4. **ESI-MS**

Peptides **ST-A1** and **ST-A2** and proteins **SC1m** and **SC2m** were characterized by UPLC-MS with the instrumental set up: Waters Acquity ultra performance LC E07SQD086W, Waters SQ detector and ESI interface, utilising a Acquity UPLC BEH 130 Å column (C18, 2.1x50 mm, 1.7 µm) and $H_2O$/ACN + 0.2% formic acid as eluents.

For the reconstitution studies of **SC1m** with **ST-A1** and **ST-A2**, the LC-MS analyses were carried out with the instrumental set up: Agilent HPLC 1260 Infiniti II, Agilent InfinityLab LC/MSD XT detector with Agilent Jet Stream source, using an Acquity UPLC BEH 300 Å column (C18, 2.1x50 mm, 1.7 µm) and $H_2O$/AcN + 0.2% formic acid as eluents.

## 2.5. **Spectroscopic studies**

**Stock solutions.** Stock solutions (*ca*. 0.4-2.0 mM) of **SC1m**, **SC2m**, and **ST-A3** were prepared in 20 mM HEPES buffer, 0.1 M NaCl at pH 7.4. Concentrated (*ca*. 0.4-2 mM) solutions of **ST-A1** and **ST-A2** and **AAHAWG-NH$_2$** were prepared by weight in 20 mM HEPES, 0.1 M NaCl solution at pH 7.4. Concentration of the stock solutions of Peptides and SpyCatcher were determined through the Lambert Beer equation by measuring the absorbance at 280 nm and using $\varepsilon_{Trp,\ 280nm}$ = 5690 $M^{-1}\ cm^{-1}$ and $\varepsilon_{Tyr,\ 280nm}$ = 1280 $M^{-1}\ cm^{-1}$ as contributions of each single residue to the total molar absorbance.[74-76] Stock solutions of $CuCl_2$ were prepared by weight from analytical grade metal salts and standardised by complexometric titrations with EDTA following standard protocols.[78] Aqueous HEPES buffer solutions (20 mM, pH 7.4) containing 0.1 M NaCl were prepared in doubly distilled water.

**UV-Visible titration experiments.** UV-Visible titration experiments were carried out at 298.2 K on a Thermo Scientific Evolution 260 Bio spectrophotometer or on a Cary3500 spectrophotometer provided with a Peltier thermostat, using 1 cm path length black quartz cuvettes. Samples of **SC1m**, **SC2m**, **ST-A1**, **ST-A2** or **ST-A3** were prepared in 20 mM HEPES, 0.1 M NaCl solution at pH 7.4 to obtain 0.4-1.0 mM final concentration. Spectrophotometric titrations were carried out by adding $CuCl_2$ to sample solutions up to ligand:metal ratio of 4-6 for SpyCatcher, and 1.25 for SpyTag peptides, respectively. Spectrophotometric titrations of reconstituted ST/SC proteins (1:1 ST/SC ratio) were performed by adding $CuCl_2$ up to 4-6 metal equiv. to a solution containing SC and ST in 1:1 ratio, incubated for 15 min at room temperature prior to titration ($C_{SpyCatcher}$ = 0.25-0.50 mM). All titrations were performed in duplicate.

**Circular dichroism (CD) titration experiments.** CD titration experiments were carried out with a Jasco J1500 spectropolarimeter equipped with a Peltier thermostat at 298.2 K, using 1 cm path length black quartz cuvettes. Samples of **SC1m**, **SC2m**, **ST-A1**, **ST-A2** or **ST-A3** were prepared in 20 mM HEPES, 0.1 M NaCl solution at pH 7.4 at 0.4-1 mM final protein concentration. CD titrations by adding $CuCl_2$ to sample solutions up to ligand:metal ratio of 4-6 for SpyCatcher, and 1.25 for SpyTag peptides, respectively. CD titrations of reconstituted ST/SC proteins (1:1 ST/SC ratio) were performed by adding $CuCl_2$ up to 4-6 metal equiv. to a solution containing SC and ST in 1:1 ratio, incubated for 15 min at room temperature prior to titration ($C_{SpyCatcher}$ = 0.25-0.50 mM). All titrations were performed in duplicate.

**Fluorescence spectroscopy titration experiments.** Fluorescence emission spectra were recorded at 298.2 K with a Horiba Jobin Yvon Fluoromax 3 or an Edinburgh FLS1000

spectrofluorimeter with $\lambda_{exc}$=280 nm and $\lambda_{em}$=290-450 nm. The excitation and emission wavelength were selected in order to observe the excitation and emission of the indolic fluorophore of Trp. Spectrofluorimetric titrations of **SC1m** and **SC2m** were carried out in aqueous buffer (20 mM HEPES, 0.1 M NaCl, pH 7.4) using solutions at 10 µM concentration. Each solution was titrated with $CuCl_2$ up to a metal:ligand ratio of *ca*. 12. Competition spectrofluorimetric titrations of **ST-A1**, **ST-A2** or **ST-A3** in the presence of the **AAHAWG-Am** peptide were carried out in aqueous buffer (20 mM HEPES, 0.1 M NaCl, pH 7.4). Samples containing *ca*. 10.0 µM ST peptide and an equimolar amount of the **AAHAWG-Am** peptide were titrated with $CuCl_2$ up to a metal:ligand ratio of *ca*. 2.5-3.0. All titrations were performed in duplicate. **ST-A3** was also studied by direct spectrofluorimetric titration with $Cu^{2+}$ monitoring the tyrosine emission quenching was used ($\lambda_{exc}$=280 nm and $\lambda_{em}$=290-450 nm, $Cu^{2+}$:peptide ratio = 0-11.25, $C_{peptide}$ = 10 µM). Spectral data were treated using the HypSpec2014 software.[79,80] Competition spectrofluorimetric titrations of the reconstituted **ST-A1/SC1m** and **ST-A2/SC1m** proteins in the presence of the **AAHAWG-NH₂** peptide were carried out in aqueous buffer (20 mM HEPES, 0.1 M NaCl, pH 7.4). Samples containing 10.0 µM ST peptide and 10.0 µM **SC1m** protein were prepared by mixing in the cuvette proper amounts of the stock solutions of the two components, left to incubate for 15 minutes, and diluted to 3 mL to afford the desired 10.0 µM concentration of the reconstituted protein. All the setup for the experiment was the same as described for the titrations of the ST peptides.

**Binding constants: experiment design.** The determination of the binding constant ($K_f$) of $Cu^{2+}$ at the ATCUN site was carried out using competition titration experiments, where the competing ligand of ATCUN SpyTag (**ST-A1**, **ST-A2** and **ST-A3**) is the ATCUN **AAHAWG-$NH_2$** hexapeptide. The log $K_f$ of the latter peptide was calculated from the potentiometric speciation model published by us in ref. Perinelli et al 2020,[59] using the $K_{ap}$ tool available in the HySS2014 software.[62] The log $K_f$ value at pH 7.4 resulted 13.5, which is in the range of conditional binding constants reported for $Cu^{2+}$/ATCUN complexes.[53,54] The quenching of luminescence data associated to the addition of $Cu^{2+}$ to the peptide is reported in Figure 4 C and D (data available from the author).

The determination of the binding constant ($K_f$) of $Cu^{2+}$ at the ATCUN site of the SpyTag peptides **ST-A1**, **ST-A2** and **ST-A3** (Table 2) was performed through competition fluorescence titration experiments. Titrations were carried out by titrating a solution containing both the SpyTag peptide and **AAHAWG-$NH_2$** in equimolar concentrations with $Cu^{2+}$ (see above for details, and Figures 4E, S5A and S8A for spectral dataset). The spectra dataset show, consistently, a plateau in the luminescence values at 350 nm for 2 eq. of $Cu^{2+}$ added, consistent with the complete saturation of ATCUN sites of both peptides (Figures 4F, S5B and S8B).

The determination of the binding constant ($K_f$) of $Cu^{2+}$ at the N-terminus of **SCm1** and **SCm2** (Table 2) was carried out by direct fluorescence titration of the proteins with $Cu^{2+}$, following the quenching of the emission of the Trp residue at 350 nm (Figure S10 and Figures 5 C and D).

For the determination of the $K_f$ of binding of the first eq. of $Cu^{2+}$ to the ATCUN sites in the reconstituted **ST-A1/SC1m** and **ST-A2/SC1m** proteins we used the same setup described here above for **ST-A1** and **ST-A2** (competition fluorescence titration) but using reconstituted ST/SC proteins in place of the ST peptides. Representation of the spectra dataset is given in Figure S15 and S16. Same data treatment was carried out on spectrofluorimetric data for the titration of **ST-A1/SC1m** and **ST-A2/SC1m** with $Cu^{2+}$. From these datasets, $K_f$ of binding of $Cu^{2+}$ to the ATCUN sites in the reconstituted **ST/SC** protein resulted 14.5(1) and 13.7(2), respectively.

For the determination of the $K_f$ of binding of the second eq. of $Cu^{2+}$ to the ATCUN sites the reconstituted **ST-A1/SC1m** and **ST-A2/SC1m** proteins we have treated the spectra dataset of the UV-Visible titration of the reconstituted proteins with $Cu^{2+}$ (Figures S12 A and D). The $K_f$ of binding of $Cu^{2+}$ to the ATCUN sites as fixed parameters (details are given below). From these datasets, $K_f$ of binding of $Cu^{2+}$ to the N-terminus of the reconstituted **ST/SC** protein resulted 3.3(1) and 3.4(1), respectively

**Binding constants: data treatment and calculations.**

All spectroscopic titration experiments were performed in triplicate, on independently prepared samples. The emission intensities recorded in competition experiments using the **AAHAWG-NH$_2$** peptide (see Figure 4E-F and Figure S5) and the absorbances recorded in direct titration experiments (see. Figure S2 and S4) were treated as a full dataset in the range 320-420 and 450-700 nm, respectively. Data treatment was carried out using the HypSpec 2014 software which allows to treat absorbance or emission at multiple wavelengths simultaneously, and to refine simultaneously multiple formation constants of metal complexes using a least-square regression procedure.[79,80] For each system studied, data from different titrations were treated together.

Affinities of SpyTag peptides for $Cu^{2+}$ were studied by competition fluorescence titrations of SpyTag peptides (ST = **ST-A1**, **ST-A2** and **ST-A3**) with the **AAHAWG-NH$_2$** peptide (P). Best fit of the spectra dataset was obtained taking into account the formation of 1:1 copper/peptide adducts (charges omitted):

$$Cu + P = [Cu(P)]$$

$$Cu + ST = [Cu(ST)]$$

The logarithm of $K_f$ of the first formation equilibrium was used as a fixed parameter (log $K_f$ = 13.5, ref. Perinelli et *al* 2020 [59]). The refined log $K_f$ values for the second equilibrium are reported in Table 2.

Affinities of SpyCatcher proteins for $Cu^{2+}$ were studied by direct fluorescence titrations of SpyCatcher proteins (SC = **SC1m** and **SC2m**). Best fit of the spectra dataset was obtained taking into account the formation of 1:1 copper/protein adducts (charges omitted):

$$Cu + SC = [Cu(SC)]$$

The refined log $K_f$ values for this equilibrium are reported in Table 2.

Affinities of reconstituted Spy proteins (Spy = **ST-A1/SC1m** or **ST-A2/SC1m**) for $Cu^{2+}$ were studied by competition fluorescence titrations with the **AAHAWG-NH₂** peptide (P) and with direct visible absorption titrations. The two spectra dataset were treated independently to obtain the $K_f$ of binding of $Cu^{2+}$ to the ATCUN sites (first eq. of $Cu^{2+}$) and to the N-termini (second eq. of $Cu^{2+}$), respectively.

The spectral dataset was treated similarly to that of the spectrofluorimetric titration of **ST-A1**, **ST-A2** and **ST-A3** with $Cu^{2+}$, as reported above. Fit of the fluorescence competition spectra dataset was obtained taking into account the formation of 1:1 copper/peptide adducts (charges omitted):

$$Cu + P = [Cu(P)]$$

$$Cu + Spy = [Cu(Spy)]$$

The logarithm of the first formation equilibrium was used as a fixed parameter (log $K$ = 13.5, ref. Perinelli et al 2020[59]). The refined log $K_f$ values for the second equilibrium resulted 14.5(1) and 13.7(2) for **ST-A1/SC1m** or **ST-A2/SC1m**, respectively.

The fit of the direct visible titration dataset was obtained considering the formation of 1:1 and 2:1 copper/Spy adducts (charges omitted):

$$Cu + Spy = [Cu(Spy)]$$

$$2\ Cu + Spy = [Cu_2(Spy)]$$

The logarithm of $K_f$ of the first formation equilibrium determined from competition fluorescence titrations was used as a fixed parameter (log $K_{f1}$ = 14.5(1) and 13.7(2) for **ST-A1/SC1m** or **ST-A2/SC1m**, respectively). The refined log $\beta$ values for the second equilibrium resulted 17.8(1) and 17.1(2) for **ST-A1/SC1m** or **ST-A2/SC1m**, respectively. These constants correspond to log $K_{f2}$ of 3.3(1) and 3.4(2) for the binding of $Cu^{2+}$ at the N-terminus of **ST-A1/SC1m** or **ST-A2/SC1m**, respectively (log $K_{f2}$ = log $\beta$ – log $K_{f1}$).

All spectra datasets were treated using the speciation models reported above. Data treatment was carried out using the HypSpec2014 software that allowed to treat all the equilibria simultaneously. In all data treatment, the spectra dataset from independent experiments were treated together. The log $K_f$ of $Cu^{2+}$/HEPES adduct at pH 7.4 was taken into account in the calculations (log $K_f$ = 2.9, calculated using HySS from data reported in ref.[81]).

The selection of the equilibria and the species to be included in the speciation model was made by a first evaluation, where clearly present, of the equivalence points (see experiments in Figures 4, 5, 7, S2, S4, S5, S8, S13, S14, S15, S16, S17). Inclusion of species with different

$Cu^{2+}$/ligand stoichiometries was attempted, in particular including $Cu^{2+}$/peptide(protein) 1:2 or 2:1 adducts, where not already present in the model. All species not included in the final models presented in this work were excluded due to either inconsistency with equivalence points or rejection during data analysis (e.g. lack of least-squares convergence or failure to pass the ANOVA Mandel test).

**ROS Formation.** Fluorescence emission spectra were recorded at 298.2 K with an Edinburgh FLS1000 spectrofluorimeter with $\lambda_{exc}$=485 nm and $\lambda_{em}$=495-650 nm. The emission at λ=525 nm was plotted as a function of time at room temperature. The samples were prepared from stock solutions of peptide **ST-A1** and SpyCatcher **SC1m** diluted to 10.0 μM and $Cu^{2+}$ diluted to 9.0 μM, in aqueous buffer (20 mM HEPES, 0.1 M NaCl, pH 7.4). $CuCl_2$ was added as 0.9 equivalents to ensure copper ion was completely complexed. Solutions were prepared using final concentrations of 1 mM $H_2O_2$, 1 mM ascorbate and 100 μM 2,7-dichlorofluorescein diacetate (DCFH-DA). DCFH-DA fluorescent dye was used to measure ROS formation in presence of ST or SpyCatcher only or in presence of SpyTag/SpyCatcher complex.

**Structural and computational studies.**

The high-confidence structures of SpyCatcher and SpyTag proteins and peptides reported in this study were predicted with the ColabFold implementation of the neural network-based, deep-learning modelling tool AlphaFold2. [82]

The analysis of protein models was carried out using *PyMOL*.[83]] Mutations on **ST-A3** were identified by rational design and their effect on the protein structure verified by AlphaFold 2 prediction software.[82]

## 3. Supporting Information Figures

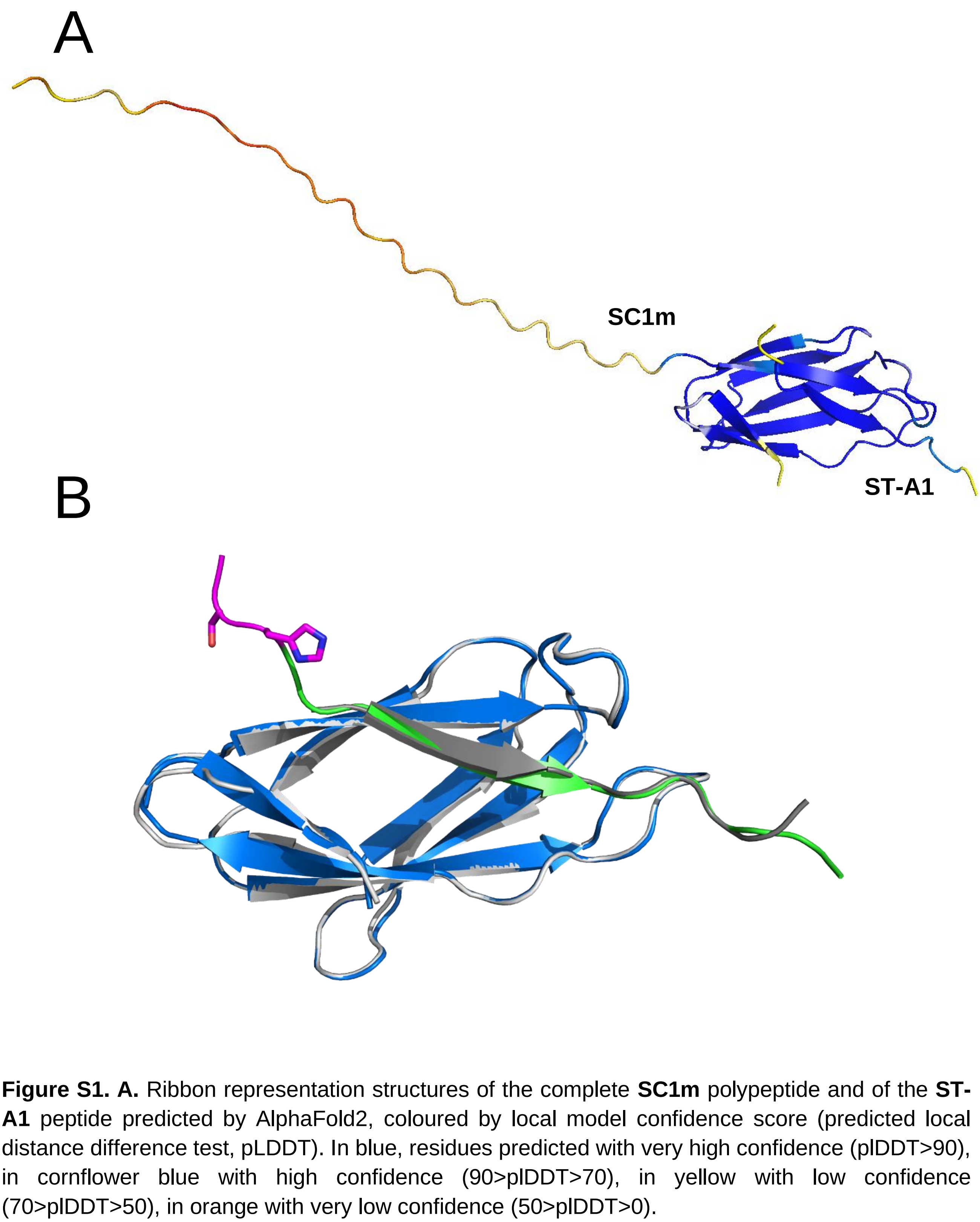


**Figure S1. A.** Ribbon representation structures of the complete **SC1m** polypeptide and of the **ST-A1** peptide predicted by AlphaFold2, coloured by local model confidence score (predicted local distance difference test, pLDDT). In blue, residues predicted with very high confidence (plDDT>90), in cornflower blue with high confidence (90>plDDT>70), in yellow with low confidence (70>plDDT>50), in orange with very low confidence (50>plDDT>0).
**B.** Overlay of the ST-A1/SC1m ribbon structures predicted by AlphaFold2 (SC1m in blue, ST-A1 in green with the ATCUN site residues in cornflower blue) with the SpyCatcher001 (light gray) and SpyTag (dark gray) crystal structure (PDB 4MLI). The low confidence model N-terminal residues (1-32) of the predicted SC1m protein are omitted.

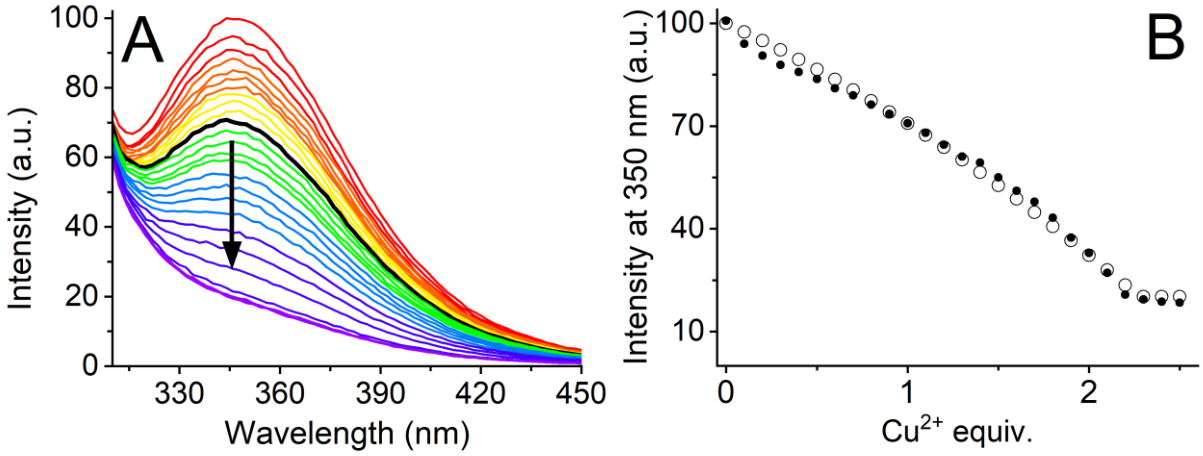


**Figure S2.** A: Fluorescence emission spectra for the titration of a solution of **ST-A1** up to 2.6 eq. of $Cu^{2+}$ ($C_{ST}$= 10 µM, 20 mM HEPES, 0.1 M NaCl, pH 7.4). $Cu^{2+}$: peptide = 0 (red spectrum) to 2.6 (purple spectrum) with 0.1 eq. of $Cu^{2+}$ additions. Spectrum for 1 eq. of $Cu^{2+}$ (vs. SpyTag) is depicted in black. B: Plot of the intensities at 350 nm vs. copper(II) equivalents for the titration dataset. Filled circles: observed; open circles: calculated.

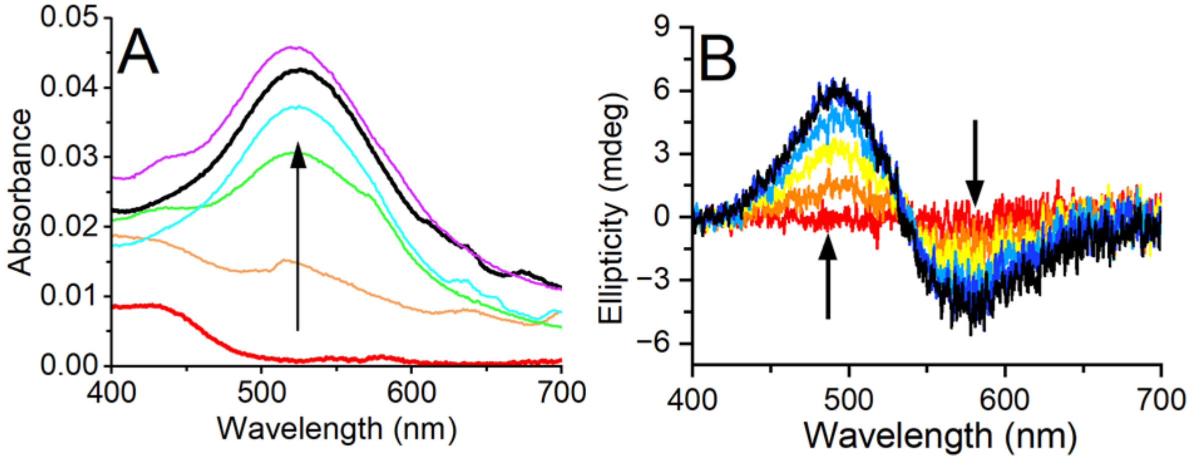


**Figure S3.** A: Visible absorption spectra for the titration of **ST-A2** with $Cu^{2+}$ ($C_{ST}$ = 400 µM, 20 mM aqueous HEPES buffer, 0.1 M NaCl solution, pH 7.4). $Cu^{2+}$: peptide = 0 (red spectrum) to 1.2 (purple spectrum), with 0.2 eq. additions. B: CD spectra for the titration **ST-A2** with $Cu^{2+}$ (same conditions as in A). $Cu^{2+}$: peptide = 0 (red spectrum) to 1.25 (purple spectrum), with 0.25 eq. additions.

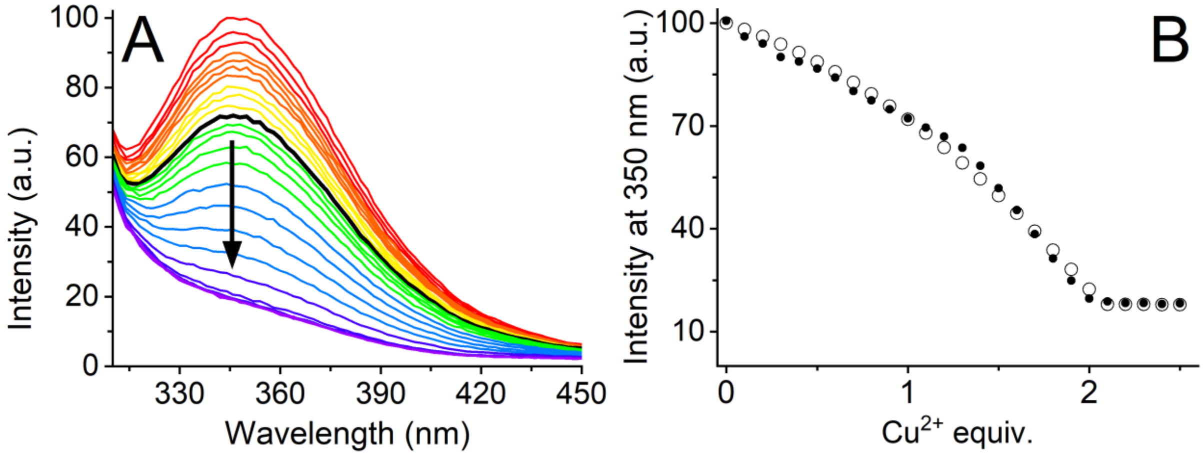


**Figure S4.** A: Fluorescence emission spectra for the titration of a solution of **ST-A2** up to 2.6 eq. of $Cu^{2+}$ ($C_{ST}$= 10 µM, 20 mM HEPES, 0.1 M NaCl, pH 7.4). $Cu^{2+}$: peptide = 0 (red spectrum) to 2.6 (purple spectrum) with 0.1 eq. of $Cu^{2+}$ additions. Spectrum for 1 eq. of $Cu^{2+}$ (vs. SpyTag) is depicted in black. B: Plot of the intensities at 350 nm vs. copper(II) equivalents for the titration dataset. Filled circles: observed; open circles: calculated.

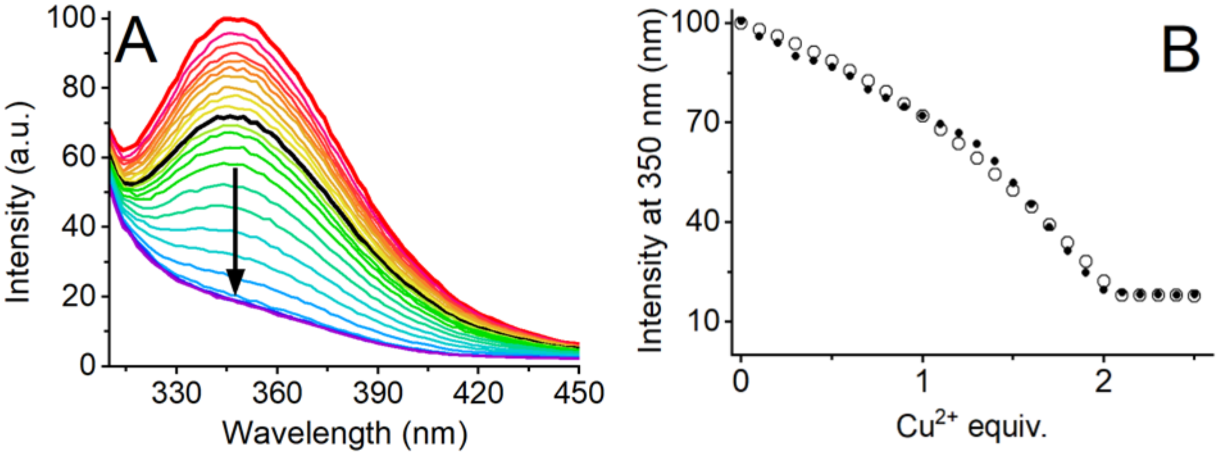


**Figure S5.** Fluorescence emission spectra (A) and intensity of emission at 350 nm in function of $Cu^{2+}$ equivalents (B) for **ST-A2** and **AAHAWG-NH$_2$** with up to 2.5 eq. of $Cu^{2+}$ ($C_{ST}$ = $C_{AAH}$ = 10 µM, 20 mM HEPES, 0.1 M NaCl, pH 7.4). $Cu^{2+}$: peptide = 0 (red spectrum) to 2.5 (purple spectrum), with 0.1 eq. additions. The spectra for 1 eq. of $Cu^{2+}$ (vs. SpyTag) are depicted in black. Filled circles: observed; open circles: calculated.

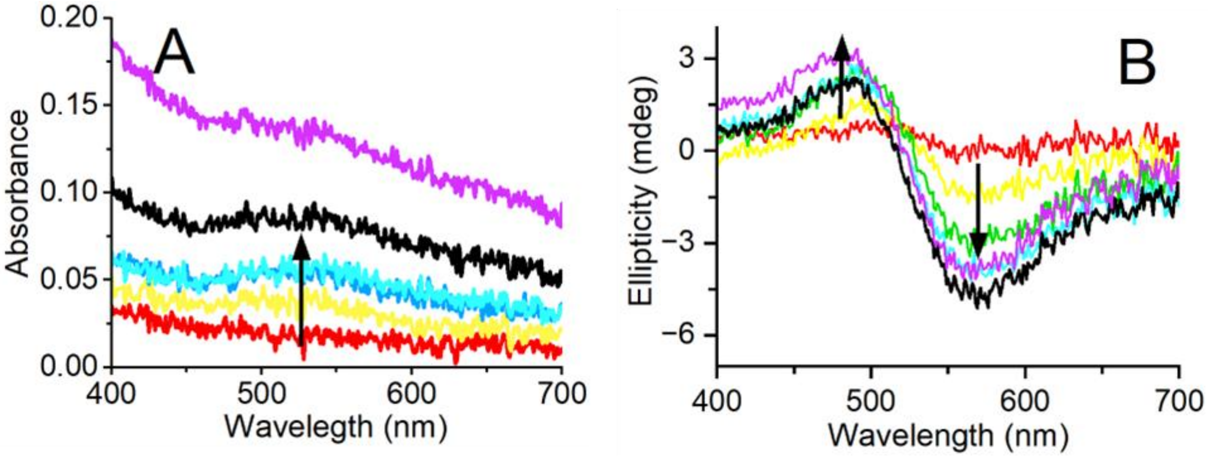


**Figure S6.** A: Visible absorption spectra for the titration of **ST-A3** with $Cu^{2+}$ ($C_{ST}$ = 400 µM, 20 mM aqueous HEPES buffer, 0.1 M NaCl solution, pH 7.4). B: CD spectra for the titration of **ST-A3** with $Cu^{2+}$ (same conditions as in A). In all plots, $Cu^{2+}$: peptide = 0 (red spectrum) to 1.25 (purple spectrum) with 0.25 eq. of $Cu^{2+}$ additions. In all plots the spectra for 1 eq. of $Cu^{2+}$ (vs. SpyTag) are depicted in black.

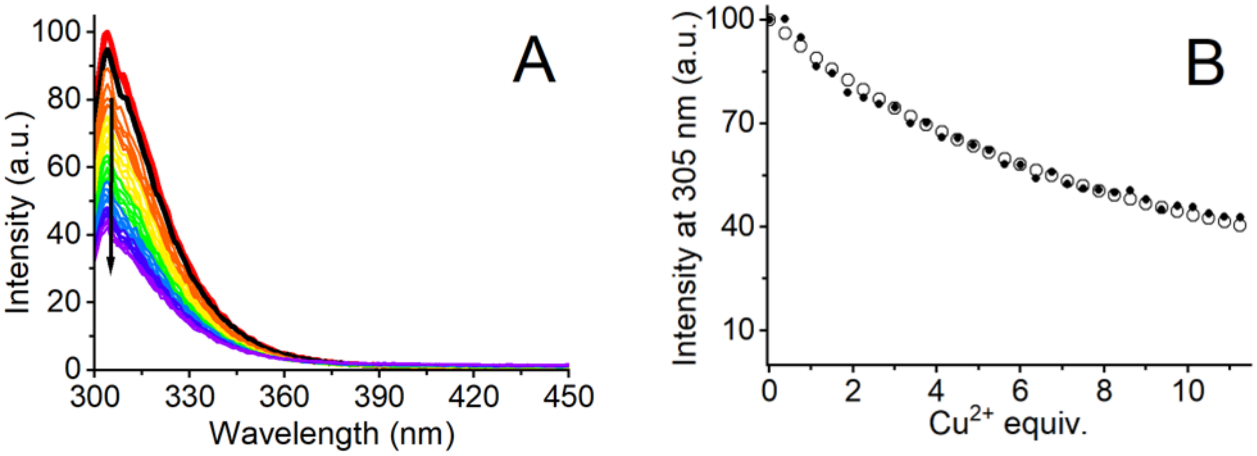


**Figure S7.** A: Fluorescence emission spectra for the titration of a solution of **ST-A3** up to 11.25 eq. of $Cu^{2+}$ ($C_{ST}$= 10 µM, 20 mM HEPES, 0.1 M NaCl, pH 7.4). $Cu^{2+}$: peptide = 0 (red spectrum) to 11.25 (purple spectrum) with 0.375 eq. of $Cu^{2+}$ additions. Spectrum for 1 eq. of $Cu^{2+}$ (vs. SpyTag) is depicted in black. B: Plot of the intensities at 305 nm vs. copper(II) equivalents for the titration dataset. Filled circles: observed; open circles: calculated.

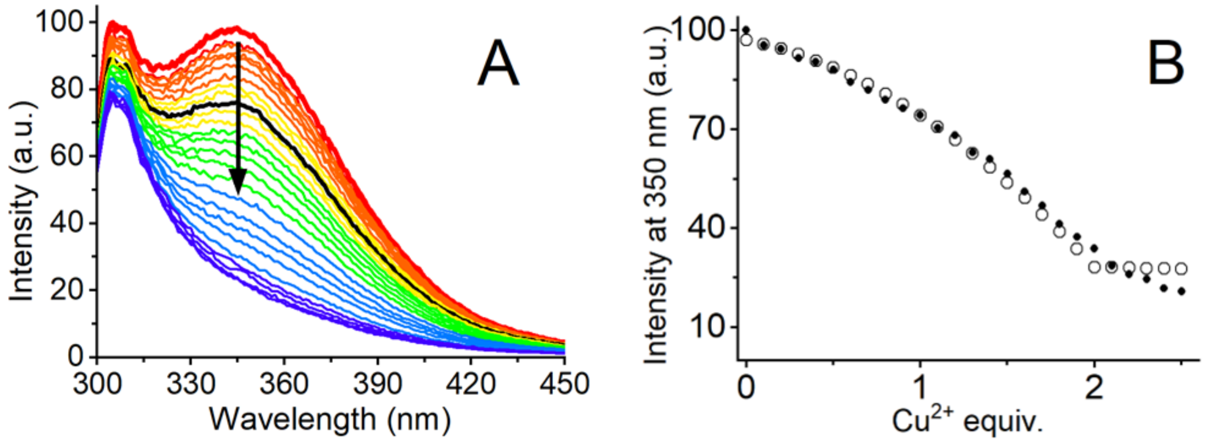


**Figure S8.** A: Normalized fluorescence emission spectra for the titration of a solution of **ST-A3** and **AAHAWG-NH$_2$** with up to 2.5 eq. of $Cu^{2+}$ ($C_{ST}$ = $C_{AAH}$ = 10.6 µM, 20 mM HEPES, 0.1 M NaCl, pH 7.4). $Cu^{2+}$: peptide = 0 (red spectrum) to 2.5 (blue spectrum), with 0.1 eq. of $Cu^{2+}$ additions. Spectrum for 1 eq. of $Cu^{2+}$ (vs. SpyTag) is depicted in black. B: Plot of the intensities at 350 nm vs. copper(II) equivalents. Filled circles: observed; open circles: calculated.

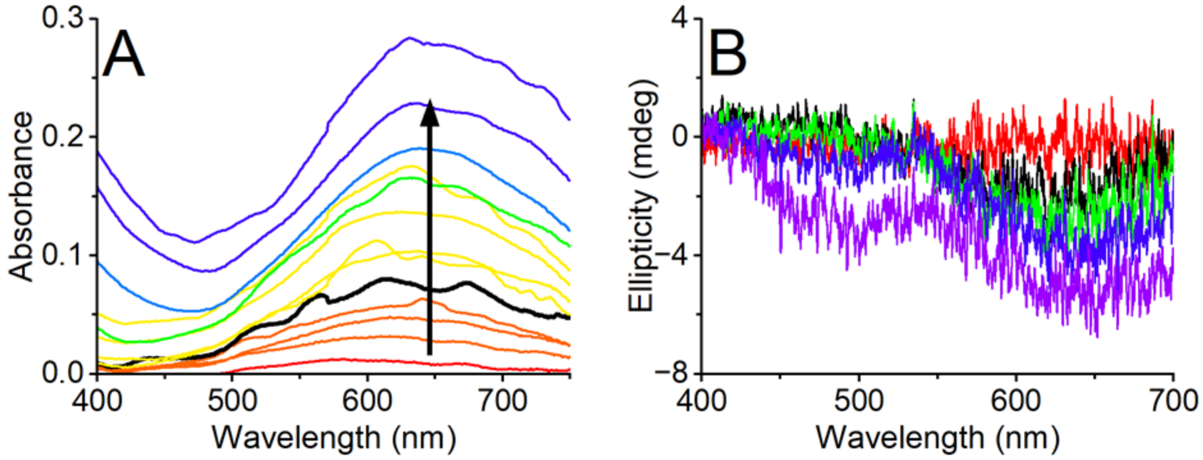


**Figure S9.** A: Visible absorption spectra for the titration of **SC1m** with $Cu^{2+}$ ($C_{ST}$ = 400 µM, 20 mM aqueous HEPES buffer, 0.1 M NaCl solution, pH 7.4). $Cu^{2+}$: **SC1m** = 0 (red spectrum) to 3.8 (purple spectrum) with 0.2 eq. of $Cu^{2+}$ additions until 1.2 eq., then with 0.4 eq. of $Cu^{2+}$ additions.
B: CD spectra for the titration of **SC1m** with $Cu^{2+}$ (same conditions as in A). $Cu^{2+}$: **SC1m** = 0 (red spectrum), 1.5 (light green spectrum), 3 (blue spectrum), 5 (purple spectrum). In all plots the spectra for 1 eq. of $Cu^{2+}$ (vs. SpyCatcher) are depicted in black.

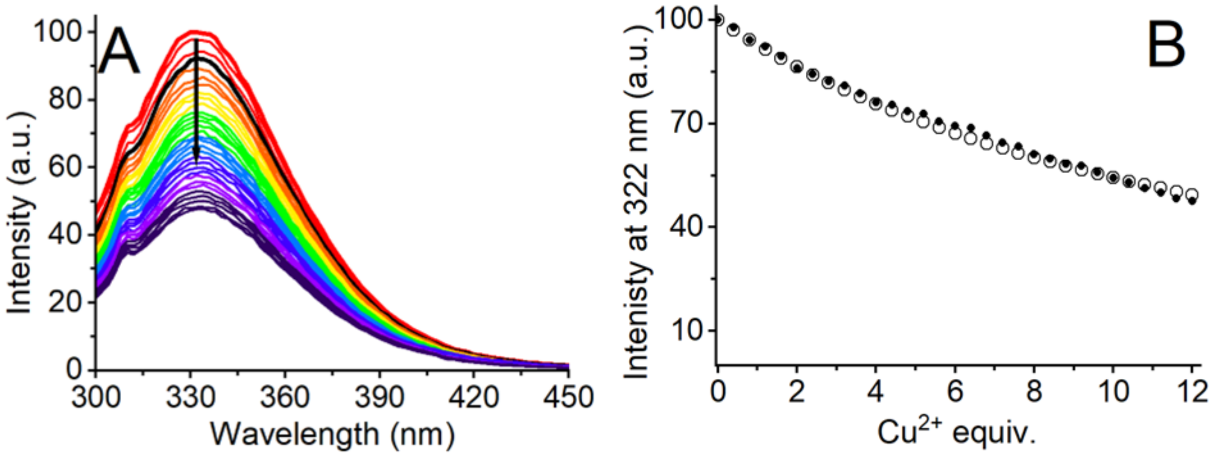


**Figure S10.** A: Fluorescence emission spectra for the titration of a solution of **SC1m** up to 12 eq. of $Cu^{2+}$ ($C_{SC1m}$= 10 μM, 20 mM HEPES, 0.1 M NaCl, pH 7.4). $Cu^{2+}$: peptide = 0 (red spectrum) to 11.25 (purple spectrum), with 0.4 eq. of $Cu^{2+}$ additions. Spectrum for 1.2 eq. of $Cu^{2+}$ (vs. SpyCatcher) is depicted in black. B: Plot of the intensities at 322 nm vs. copper(II) equivalents for the titration dataset. Filled circles: observed; open circles: calculated.

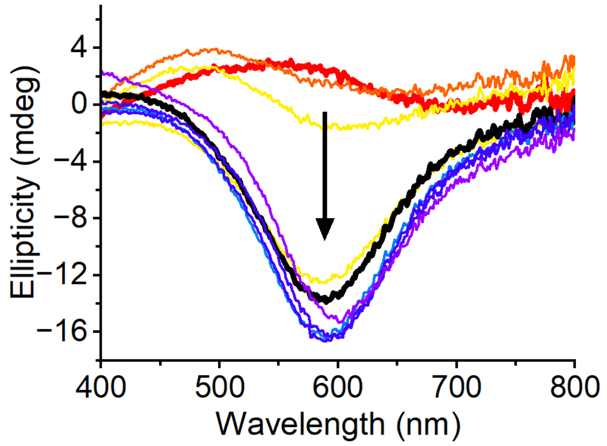


**Figure S11.** CD spectra for the titration of **SC2m** with $Cu^{2+}$ ($C_{SC}$ = 948 μM, 20 mM aqueous HEPES buffer, 0.1 M NaCl solution, pH 7.4). $Cu^{2+}$: protein = 0 (red spectrum) to 3 (purple spectrum), with 0.25 eq. additions

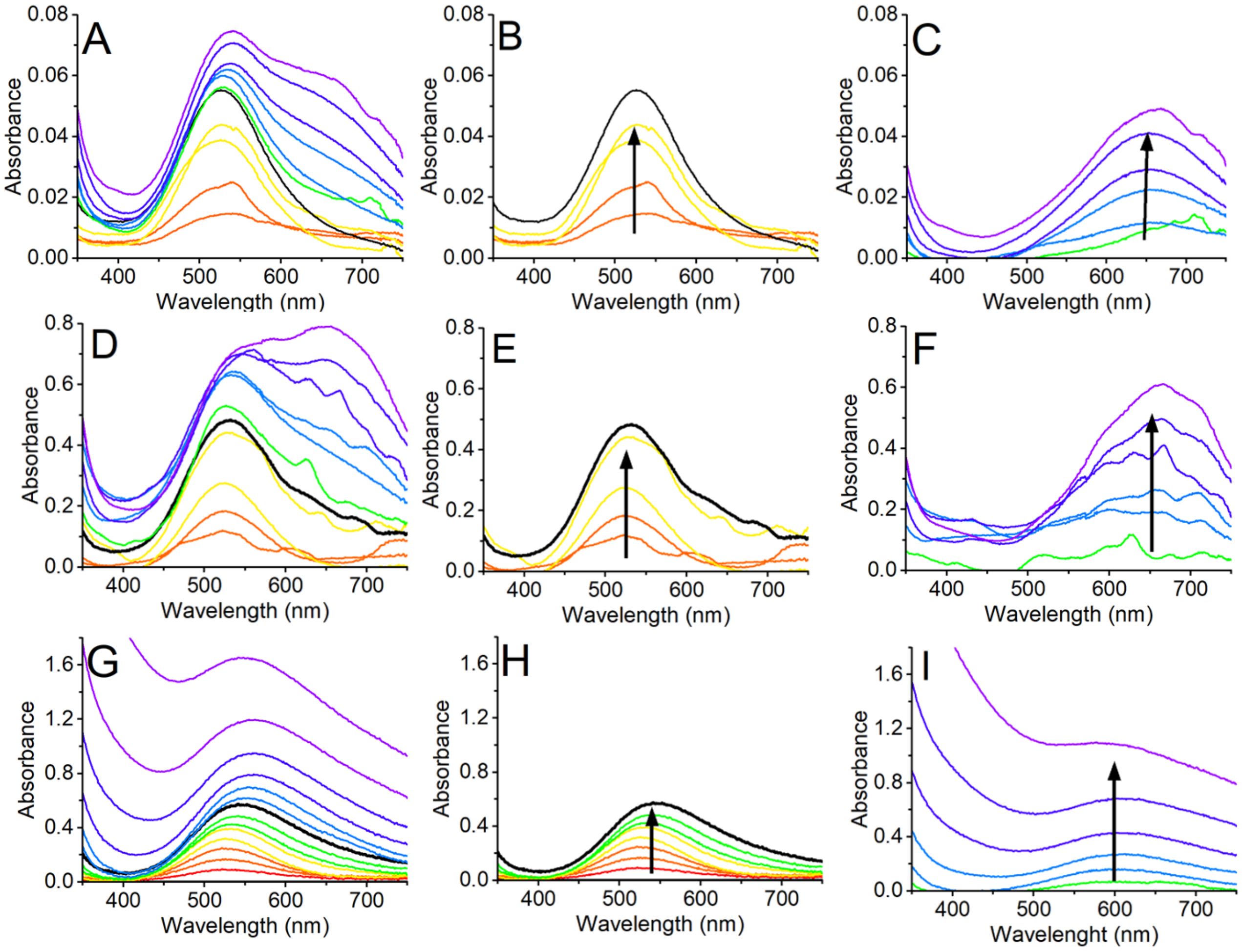


**Figure S12.** Visible absorption spectra data set for the titration of **ST-A1/SC1m** (A) **ST-A2/SC1m** (D) and **ST-A3/SC2m** (G) with $Cu^{2+}$ ($C_{SC}$= $C_{ST}$=400 µM, 20 mM aqueous HEPES buffer, 0.1 M NaCl solution, pH 7.4). Difference spectra obtained subtracting the 0 equivalent spectra. For **ST-A1/SC1m** (A) and **ST-A2/SC1m** (D) $Cu^{2+}$:**ST** = 0 (orange spectrum) to 2.2 (purple spectrum) with 0.2 eq. of $Cu^{2+}$ additions. For **ST-A3/SC2m** (G) $Cu^{2+}$:**ST** = 0 (orange spectrum) to 2.5 (purple spectrum) with 0.125 eq. of $Cu^{2+}$ additions. In all plots, spectra for 1 eq. of $Cu^{2+}$ are represented in black.

Difference absorption spectra for the titration of **ST-A1/SC1m** (B) **ST-A2/SC1m** (E) and **ST-A3/SC2m** (H) with $Cu^{2+}$ until 1 equivalent of $Cu^{2+}$ (bald black). Difference spectra were obtained subtracting the spectrum at 0 equivalents of $Cu^{2+}$.

Difference absorption spectra for the titration of **ST-A1/SC1m** (C) **ST-A2/SC1m** (F) and **ST-A3/SC2m** (I) with $Cu^{2+}$ from 1.2 equivalent of $Cu^{2+}$. Difference spectra were obtained subtracting the spectrum at 1 equivalent of $Cu^{2+}$.

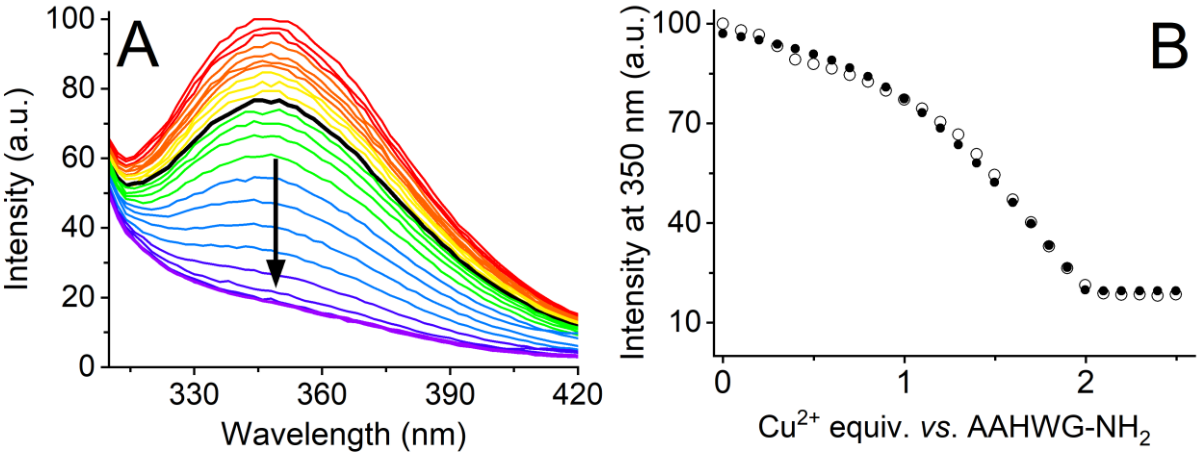


**Figure S13.** Fluorescence emission spectra for the titration of a solution of **ST-A1/SC1m** and **AAHAWG-NH₂** with $Cu^{2+}$ (A) and plot of the intensity values at 350 nm (B) ($C_{ST/SC}$= $C_{AAHAWG-NH2}$ = 10 µM, 20 mM HEPES, 0.1 M NaCl, pH 7.4). $Cu^{2+}$: **AAHAWG-NH₂** = 0 (red spectrum) to 2.5 (purple spectrum), with 0.1 eq. of $Cu^{2+}$ additions. Spectrum for 1.0 eq. of $Cu^{2+}$ (vs. **AAHAWG-NH₂**) is depicted in black. B: Plot of the intensities at 350 nm vs. copper(II) equivalents for the titration dataset. Open circles: observed; filled circles: calculated.

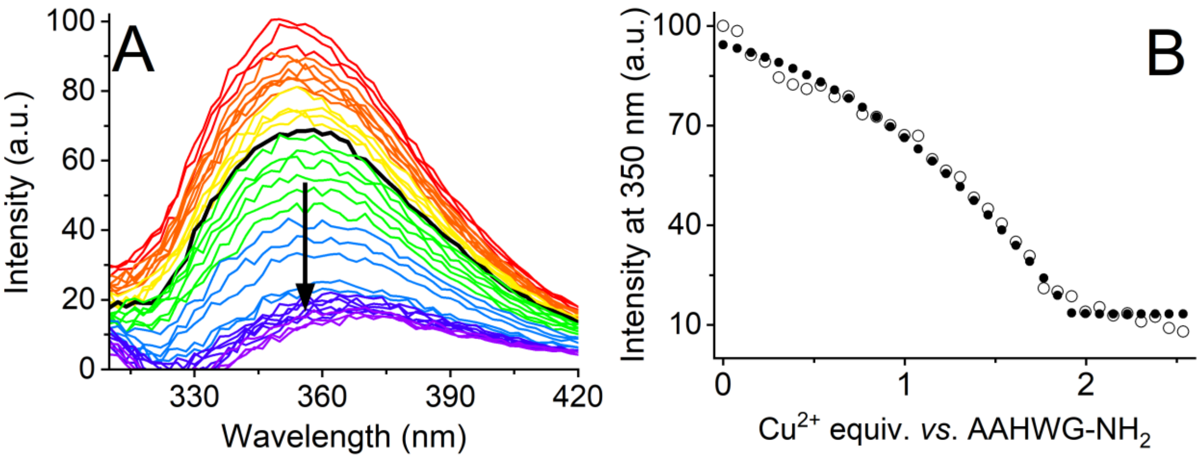


**Figure S14.** Fluorescence emission spectra for the titration of a solution of **ST-A2/SC1m** and **AAHAWG-NH₂** with $Cu^{2+}$ (A) and plot of the intensity values at 350 nm (B) ($C_{ST/SC}$= $C_{AAHAWG-NH2}$ = 10 µM, 20 mM HEPES, 0.1 M NaCl, pH 7.4). $Cu^{2+}$: **AAHAWG-NH₂** = 0 (red spectrum) to 2.5 (purple spectrum), with 0.1 eq. of $Cu^{2+}$ additions. Spectrum for 1.0 eq. of $Cu^{2+}$ (vs. **AAHAWG-NH₂**) is depicted in black. B: Plot of the intensities at 350 nm vs. copper(II) equivalents for the titration dataset. Open circles: observed; filled circles: calculated.

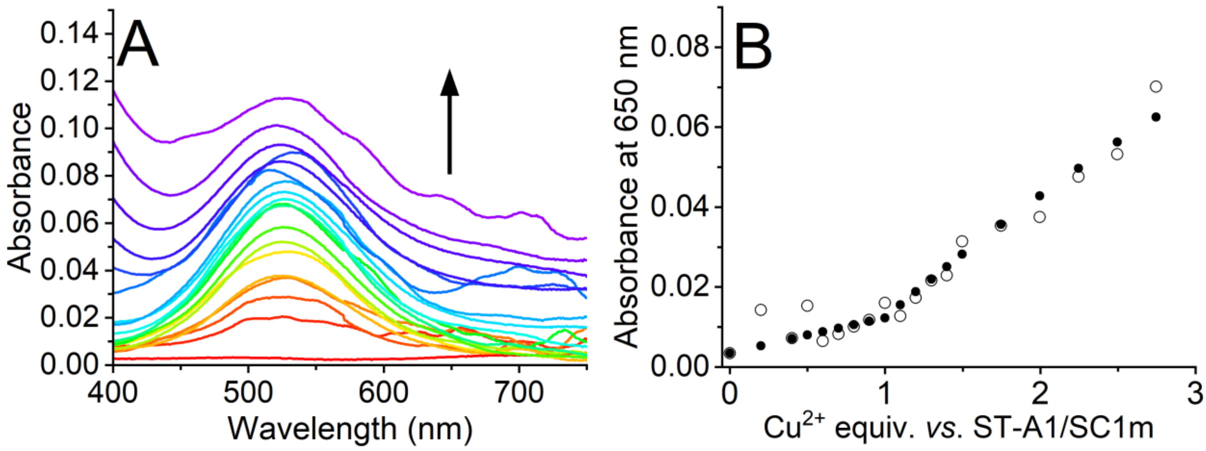


**Figure S15.** Visible absorption spectra data set for the titration of **ST-A1/SC1m** with $Cu^{2+}$ (A) and plot of the absorbance values at 350 nm (B) ($C_{ST/SC}$= 400 µM, 20 mM aqueous HEPES buffer, 0.1 M NaCl solution, pH 7.4). $Cu^{2+}$: **ST-A2/SC1m** = 0 (orange spectrum) to 2.75 (purple spectrum) with 0.2-0.4 eq. of $Cu^{2+}$ additions.

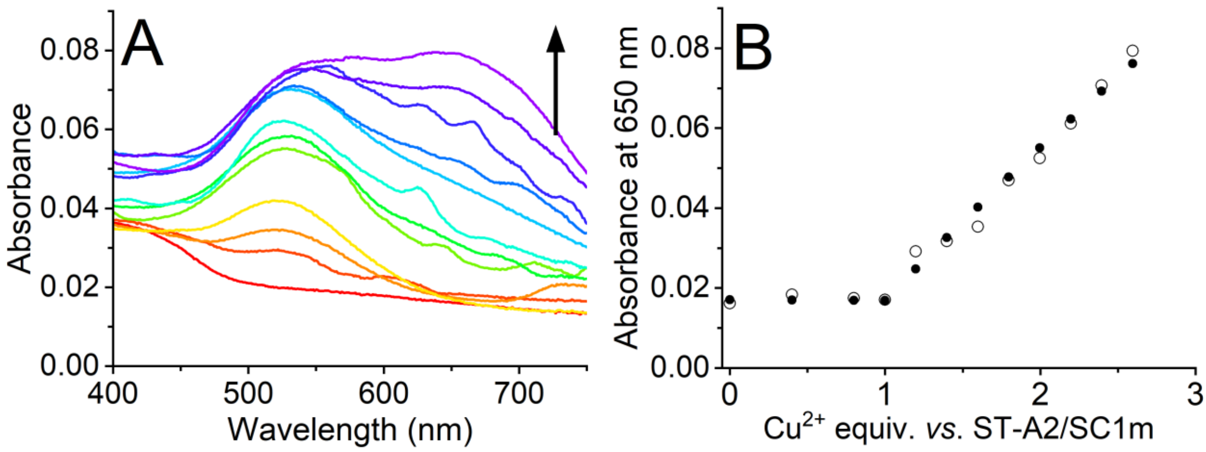


**Figure S16.** Visible absorption spectra data set for the titration of **ST-A2/SC1m** with $Cu^{2+}$ (A) and plot of the absorbance values at 350 nm (B) ($C_{ST/SC}$= 400 µM, 20 mM aqueous HEPES buffer, 0.1 M NaCl solution, pH 7.4). $Cu^{2+}$: **ST-A2/SC1m** = 0 (orange spectrum) to 2.6 (purple spectrum) with 0.4 eq. of $Cu^{2+}$ additions.

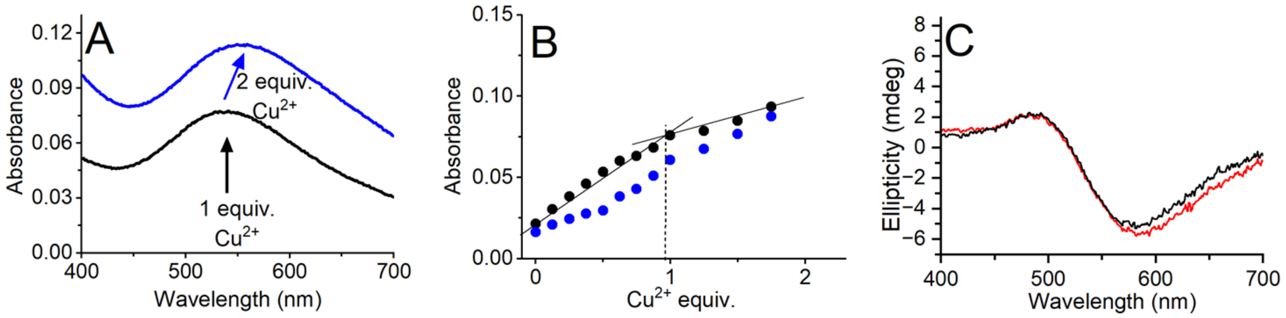


**Figure S17.** A: Visible molar absorption spectra of **ST-A3/SC2m** with 1 equivalent of $Cu^{2+}$ (black) and 2 equivalent of $Cu^{2+}$ (blue). ($C_{SC2m}$= $C_{ST\text{-}A3}$ = 500 µM, 20 mM aqueous HEPES buffer, 0.1 M NaCl solution, pH 7.4). B: Molar absorption values for the titration of **ST-A3/SC2m** with $Cu^{2+}$ at 535 nm (black) and 600 nm (blue) as a function of $Cu^{2+}$ equivalents. C: Comparison of CD spectra of **ST-A3/SC2m** (black) and **ST-A3** (red) in presence of 1 equivalent of copper each. (C= 500 µM, 20 mM aqueous HEPES buffer, 0.1 M NaCl solution, pH 7.4).

### Discussion Figure S17

UV-visible spectroscopic analysis of the **ST-A3/SC2m** adduct provided results consistent with those obtained for **ST-A1/SC1m** and **ST-A2/SC1m**. The addition of 1 $Cu^{2+}$ equivalent to **ST-A3/SC2m** resulted in an increase of the $Cu^{2+}$/ATCUN absorption band at ca. 525 nm (Figure S17A). Addition of more than 1 $Cu^{2+}$ eq. led to the appearance of a band at ca. 600 nm (see Figure S12I for representative differential spectra). Despite a baseline shift due to opalescence formation, the appearance of the latter band is fully consistent with the binding of the second equivalent of $Cu^{2+}$ to the N-term of **SC2m**. This was precisely the same effect observed analyzing the SpyCatcher component alone (Figure 5A). Finally, also the CD spectra measured after the addition of 1 $Cu^{2+}$eq. to **ST-A3/SC2m** display two bands consistent with $Cu^{2+}$ ion coordination at the ATCUN site and they are superimposable on those observed with the SpyTag **ST-A3** peptide alone (Figure S17C). Spectral analysis in the presence of $Cu^{2+}$ amounts above the 1 eq. level was however complicated by the appearance of opalescence.

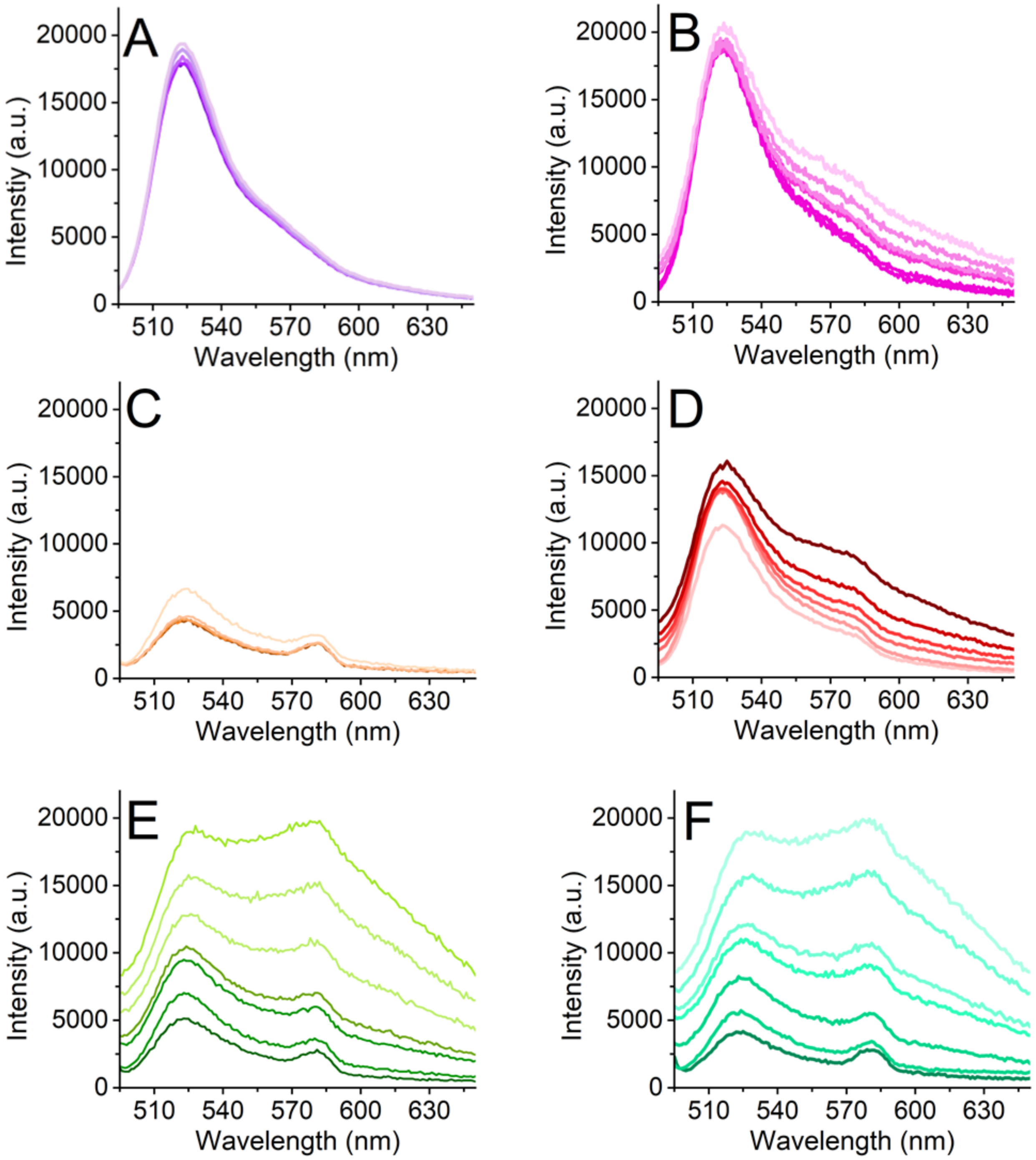


**Figure S18.** Fluorescence emission spectra for ROS formation in presence of 1 mM $H_2O_2$, 1 mM ascorbate and 100 µM 2,7-dichlorofluorescein diacetate (DCFH-DA) catalyzed by **ST-A1 (A), ST-A1:Cu (B), SC1m (C) and SC1m:Cu (D).** In panel **E** and **F** ROS formation were catalyzed by **ST/SC** complex in presence of **$Cu^{2+}$**. In panel E, $Cu^{2+}$ was added a solution of **SC1m** and **ST-A1** mixed with $H_2O_2$/Asc/DCFH-DA; in panel F $Cu^{2+}$ was incubated with **ST-A1** at least 15 min before to be added to $H_2O_2$/Asc/DCFH-DA and **SC1m** ($C_{SC1m}$= 10 µM, $C_{ST-A1}$= 10 µM, $C_{CuCl2}$= 9 µM, 25 mM HEPES, 0.1 M NaCl, pH 7.4).

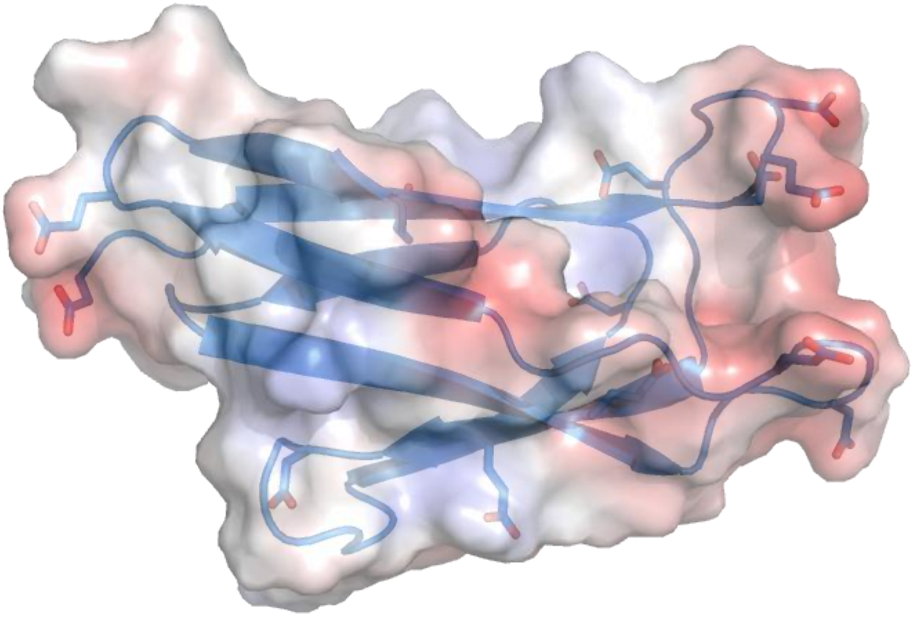

**Figure S19.** Graphic representation of the structured portion of **SC1m** (model obtained from AlphaFold2) overlapped to its electrostatic potential surface (obtained with the Electrostatic calculation tool in PyMol). Red: negatively charged regions; blue: positively charged regions. Asp and Glu residues in the sequence are represented as sticks.

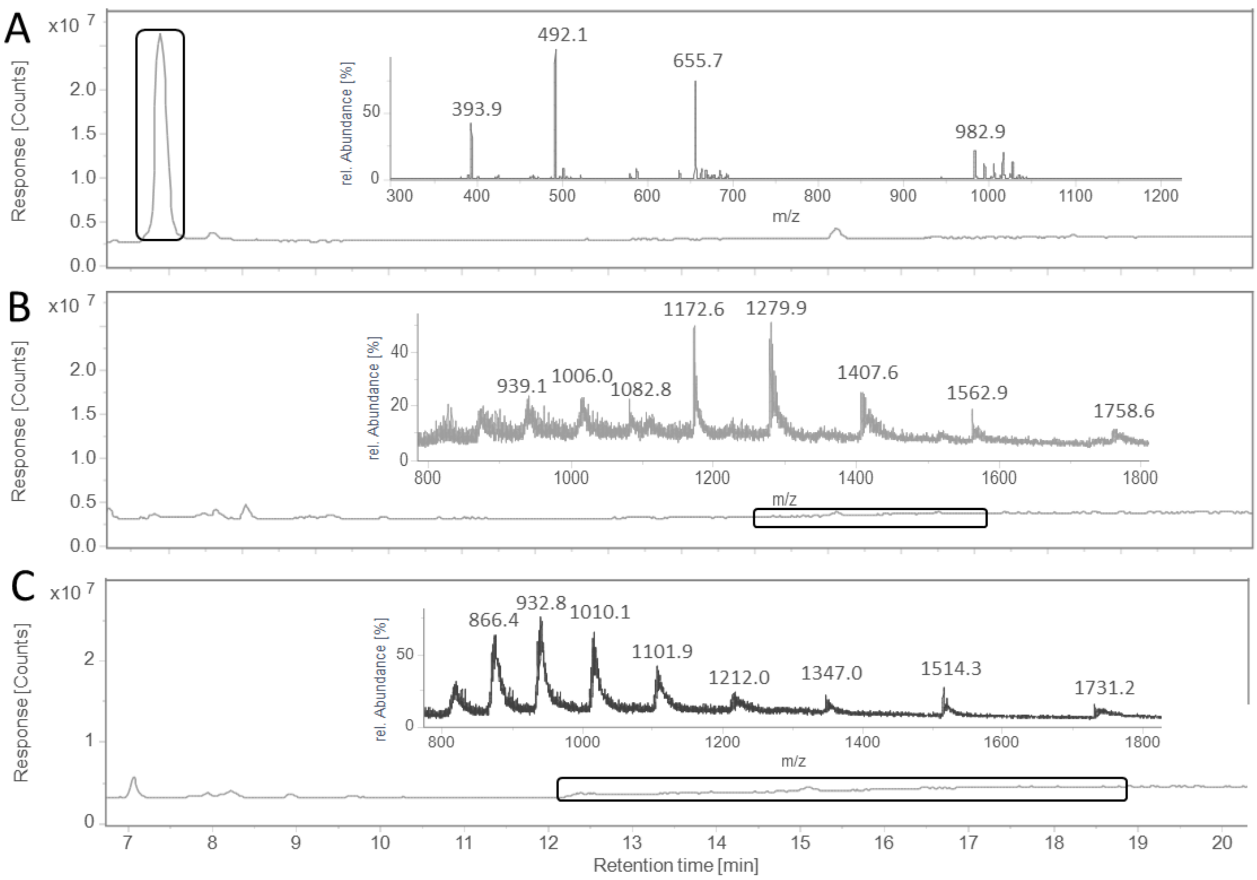


**Figure S20.** LC-MS chromatograms and mass spectra of the species for the reconstitution of **ST-A1** with **SC1m** complex.

(A) Chromatogram of a **ST-A1** sample ($C_{ST\text{-}A1}$=25 µM, 20 mM aqueous HEPES buffer, 0.1 M NaCl solution, pH 7.4). Inset: mass spectrum of the peak at retention time *ca.* 7.5 minutes. (**ST-A1** m.w.: 1964.29 g/mol. Calculated $[M+2H]^{2+}$: 983.1; $[M+3H]^{3+}$: 655.8; $[M+4H]^{4+}$: 492.1; $[M+5H]^{5+}$: 393.9. Found $[M+2H]^{2+}$: 982.9; $[M+3H]^{3+}$: 655.7; $[M+4H]^{4+}$: 492.1; $[M+5H]^{5+}$: 393.9).

(B) Chromatogram of a **ST-A1/SC1m** sample ($C_{ST\text{-}A1}$=$C_{SC1m}$=25 µM, 20 mM aqueous HEPES buffer, 0.1 M NaCl solution, pH 7.4). The sample was analysed after 5' of incubation of **SC1m** and **ST-A1**. Inset: mass spectrum of the broad band at retention time 12-19 minutes. Inset: mass spectrum of the broad band at retention time 12-19 minutes. (**ST-A1/SC1m** M.w.: 14056.56 g/mol. Found $[M+8H]^{8+}$:1758.6; $[M+9H]^{9+}$:1562.9; $[M+10H]^{10+}$:1407.6; $[M+11H]^{11+}$: 1279.9; $[M+12H]^{12+}$:1172.6; $[M+13H]^{13+}$: 1082.8; $[M+14H]^{14+}$: 1006.0; $[M+15H]^{15+}$: 939.1. Calculated $[M+8H]^{8+}$: 1758.0; $[M+9H]^{9+}$: 1562.8; $[M+10H]^{10+}$: 1406.7; $[M+11H]^{11+}$: 1278.9; $[M+12H]^{12+}$: 1172.4; $[M+13H]^{13+}$: 1082.3; $[M+14H]^{14+}$: 1005.0 $[M+15H]^{15+}$: 938.1).

(C) Chromatogram of a **SC1m** sample ($C_{SC1m}$=25 µM, 20 mM aqueous HEPES buffer, 0.1 M NaCl solution, pH 7.4). Inset: mass spectrum of the broad band at retention time 14-17 minutes. (**SC1m** M.w.: 12110.27 g/mol. m/z= Found $[M+7H]^{7+}$:1731.2; $[M+8H]^{8+}$: 1514.3 $[M+9H]^{9+}$: 1347.0; $[M+10H]^{10+}$: 1212.0; $[M+11H]^{11+}$: 1101.9; $[M+12H]^{12+}$ 1010.1; $[M+13H]^{13+}$: 932.8; $[M+14H]^{14+}$: 866.4. Calculated $[M+7H]^{7+}$:1731.0; $[M+8H]^{8+}$: 1514.0; $[M+9H]^{9+}$: 1346.6; $[M+10H]^{10+}$: 1212.0; $[M+11H]^{11+}$: 1101.9; $[M+12H]^{12+}$: 1010.2; $[M+13H]^{13+}$: 932.6; $[M+14H]^{14+}$: 866.0).

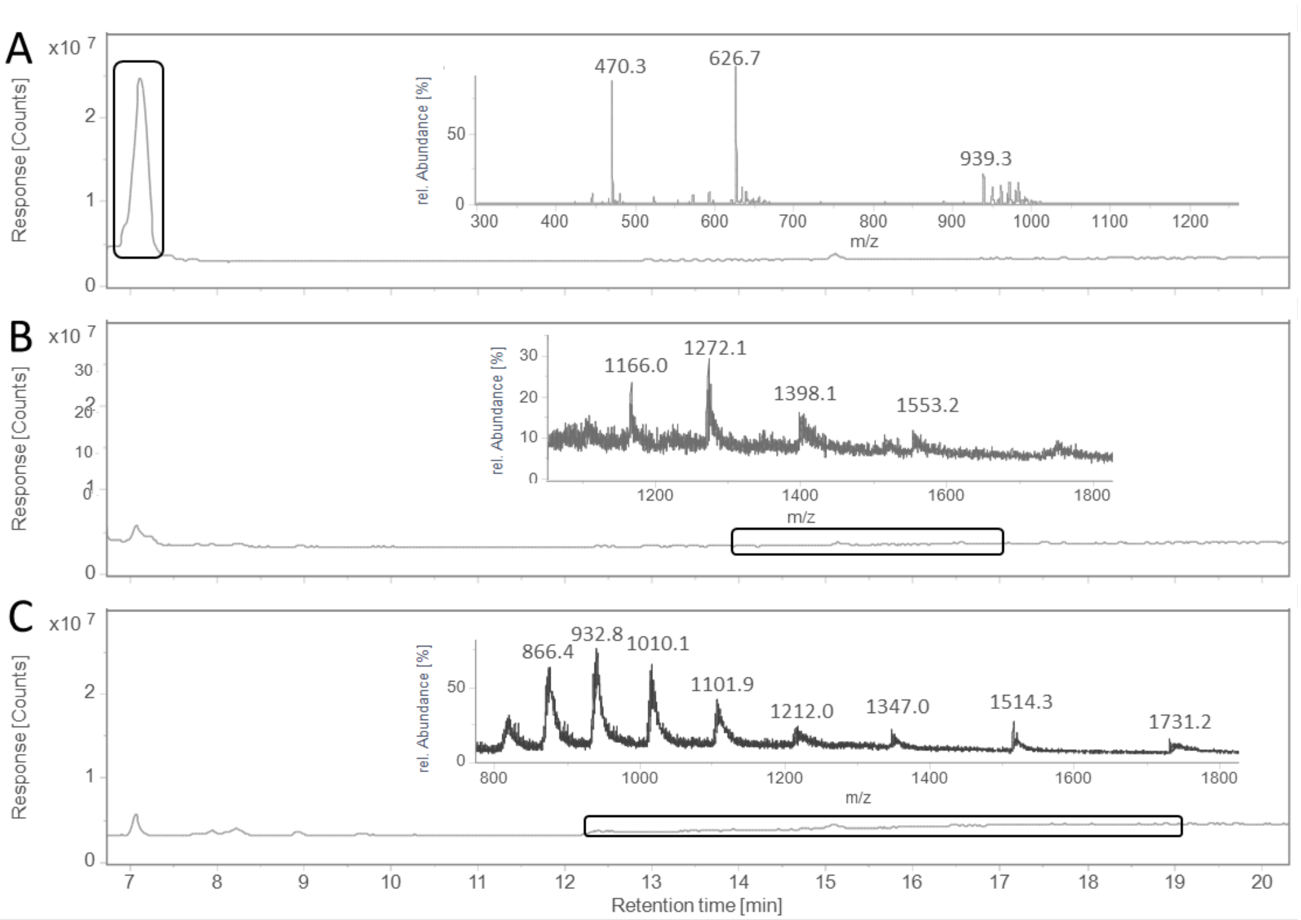


**Figure S21.** LC-MS chromatograms and mass spectra of the species for the reconstitution of **ST-A2** with **SC1m** complex**.**

(A) Chromatogram of a **ST-A2** sample ($C_{ST\text{-}A2}$=25 µM, 20 mM aqueous HEPES buffer, 0.1 M NaCl solution, pH 7.4). Inset: mass spectrum of the peak at retention time 7 minutes. (**ST-A2** M.w.: 1877.18 g/mol. Found $[M+2H]^{2+}$: 939.3; $[M+3H]^{3}$: 626.7; $[M+4H]^{4+}$: 470.3. Calculated $[M+2H]^{2+}$: 939.6; $[M+3H]^{3}$: 626.5; $[M+4H]^{4+}$: 470.3.)

(B) Chromatogram of a **ST-A2/SC1m** sample ($C_{ST\text{-}A2}$=$C_{SC1m}$=25 µM, 20 mM aqueous HEPES buffer, 0.1 M NaCl solution, pH 7.4). The sample was analysed after 5' of incubation of **SC1m** and **ST-A2**. Inset: mass spectrum of the broad band at retention time 14-17 minutes. Inset: mass spectrum of the broad band at retention time 12-19 minutes. (**ST-A2/SC1m** M.w.: 13969.35 g/mol. Found $[M+9H]^{9+}$:1553.2;$[M+10H]^{10+}$: 1398.1; $[M+11H]^{11+}$:1272.1; $[M+12H]^{12+}$: 1166.0. Calculated $[M+9H]^{9+}$: 1553.2; $[M+10H]^{10+}$: 1397.9; $[M+11H]^{11+}$: 1270.9; $[M+12H]^{12+}$ : 1165.1.)

(C) Chromatogram of a **SC1m** sample ($C_{SC1m}$=25 µM, 20 mM aqueous HEPES buffer, 0.1 M NaCl solution, pH 7.4). Inset: mass spectrum of the broad band at retention time 12-19 minutes. (**SC1m** M.w.: 12110.27 g/mol. m/z= Found $[M+7H]^{7+}$:1731.2; $[M+8H]^{8+}$: 1514.3 $[M+9H]^{9+}$: 1347.0; $[M+10H]^{10+}$: 1212.0; $[M+11H]^{11+}$: 1101.9; $[M+12H]^{12+}$ 1010.1; $[M+13H]^{13+}$: 932.8; $[M+14H]^{14+}$: 866.4. Calculated $[M+7H]^{7+}$:1731.0; $[M+8H]^{8+}$: 1514.0; $[M+9H]^{9+}$: 1346.6; $[M+10H]^{10+}$: 1212.0; $[M+11H]^{11+}$: 1101.9 ; $[M+12H]^{12+}$ : 1010.2; $[M+13H]^{13+}$: 932.6; $[M+14H]^{14+}$: 866.0).

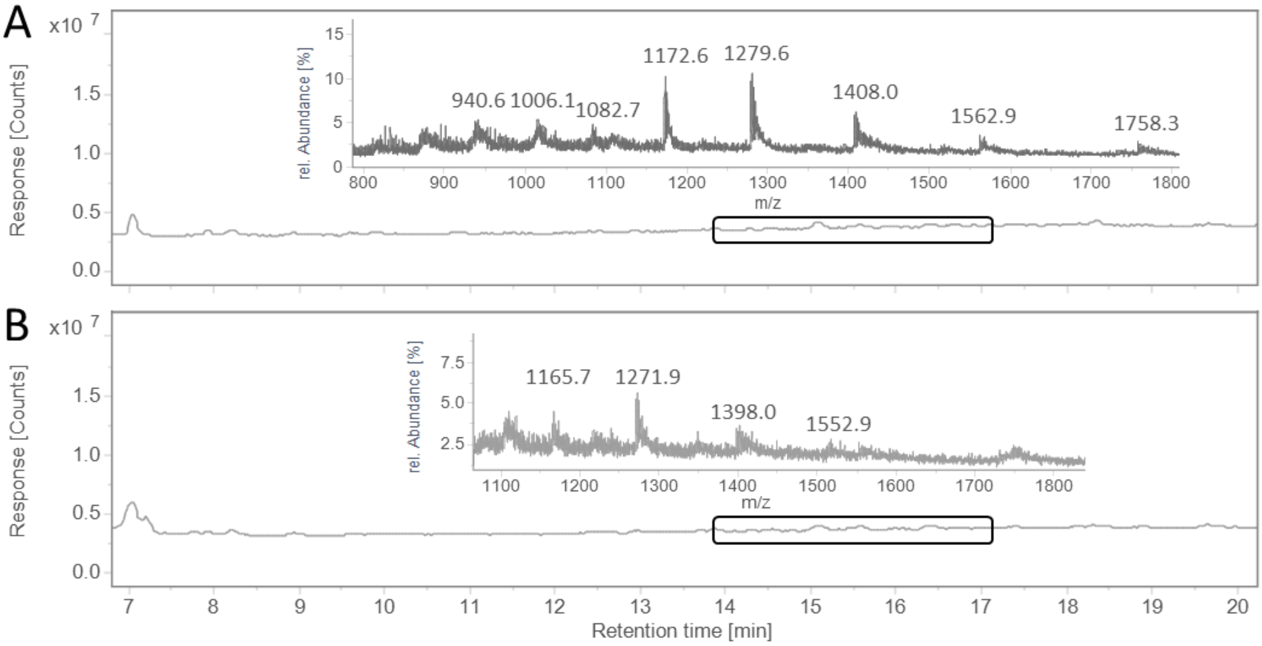


**Figure S22.** LC-MS chromatograms and mass spectra of the species for the reconstitution of **ST-A1** and **ST-A2** with **SC1m** complexes in presence and absence of $Cu^{2+}$.

Chromatograms of **ST-A1/SC1m** (A) and **ST-A2/SC1m** (B) samples with 2 equivalents of $Cu^{2+}$ ($C_{ST}$=$C_{SC1m}$=25 µM, $C_{CuCl2}$=50 µM, 20 mM aqueous HEPES buffer, 0.1 M NaCl solution, pH 7.4). The sample was prepared mixing an equal amount of **SC1m** and **ST-A1** solutions with 1 equivalent of $Cu^{2+}$ each. The sample were analysed after 5' of incubation of **SC1m** and **ST-A1**. Inset: mass spectrum of the broad band at retention time 14-17 minutes.

(**ST-A1/SC1m** M.w.: 14056.56 g/mol. Found $[M+8H]^{8+}$:1758.3; $[M+9H]^{9+}$:1562.9; $[M+10H]^{10+}$:1408.0; $[M+11H]^{11+}$: 1279.6; $[M+12H]^{12+}$:1172.6; $[M+13H]^{13+}$: 1082.7; $[M+14H]^{14+}$: 1006.1 $[M+15H]^{15+}$: 940.6. Calculated $[M+8H]^{8+}$: 1758.0; $[M+9H]^{9+}$: 1562.8; $[M+10H]^{10+}$: 1406.7; $[M+11H]^{11+}$: 1278.9; $[M+12H]^{12+}$: 1172.4; $[M+13H]^{13+}$: 1082.3; $[M+14H]^{14+}$: 1005.0 $[M+15H]^{15+}$: 938.1).

(**ST-A2/SC1m** M.w.: 13969.35 g/mol. Found $[M+9H]^{9+}$:1552.9; $[M+10H]^{10+}$: 1398.0; $[M+11H]^{11+}$:1272.9; $[M+12H]^{12+}$: 1165.7. Calculated $[M+9H]^{9+}$: 1553.2; $[M+10H]^{10+}$: 1397.9; $[M+11H]^{11+}$: 1270.9; $[M+12H]^{12+}$: 1165.1.)

M/z ions corresponding to the $Cu^{2+}$/ST/SC adducts are absent due to the acidic conditions of the eluent used in the LC-MS analysis. ($H_2O$/ACN + 0.2% formic acid, see experimental section)

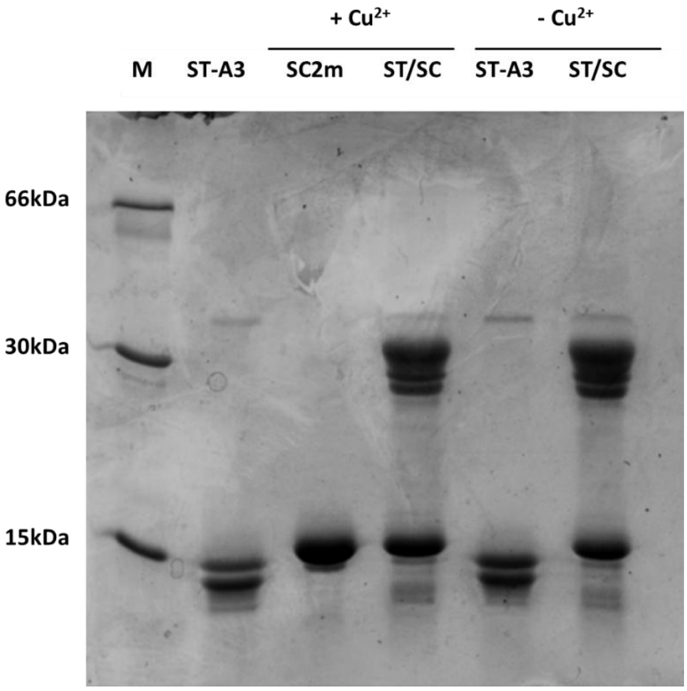


**Figure S23.** SDS-PAGE analysis of reconstitution of the **SC2m** (25 µM) and **ST-A3** (10 µM) complexes in HEPES 20 mM at pH 7.4, 0.15 M NaCl. Each protein is incubated for 5 min at room temperature in the presence (+) or absence (-) of equimolar copper(II) sulfate amounts. Samples were mixed and analyzed after 5 minutes by SDS-PAGE.

**Table S1** Nucleotide and amino acid sequences of SpyCatcher proteins and SpyTag peptides. The His-tag sequence is in dark green, in blue the TEV protease recognition sequence and in orange the Thrombin one. In red the SpyCatcher sequence and in light green the *E.coli* Thioredoxin one. The SpyTag sequence is underlined.

<table>
<tr><th>Name</th><th>Nucleotide sequence</th><th>Aminoacid sequence</th><th>Refs</th></tr>
<tr><td>SCm</td><td>CCATGGGCAGCAGCCACCACCACCACCAC<br>CACAGCGAGAACCTGTACTTCCAGAGCGG<br>CCTGGTGCCGCGCGGCAGCGGCGCCATGG<br>TAACCACCTTATCAGGTTTATCAGGTGAG<br>CAAGGTCCGTCCGGTGATATGACAACTGA<br>AGAAGATAGTGCTACCCAGATTAAATTCT<br>CAAAACGTGATGAGGACGGCCGTGAGTTA<br>GCTGGTGCAACTATGGAGTTGCGTGATTC<br>ATCTGGTAAAACTATTAGTACATGGATTT<br>CAGATGGACAGGTGAAGGATTTCTACCTG<br>TATCCAGGAAAATATACATTTGTCGAAAC<br>CGCAGCACCAGACGGTTATGAGGTAGCAA<br>CTGCTATTACCTTTACAGTTAATGAGCAA<br>GGTCAGGTTACTGTAAATTAAAAGCTT</td><td>MGSSHHHHHHSENLYFQ|SGLVPR|GSGAMV<br>TTLSGLSGEQGPSGDMTTEEDSATQIKFSKR<br>DEDGRELAGATMELRDSSGKTISTWISDGQV<br>KDFYLYPGKYTFVETAAPDGYEVATAITFTV<br>NEQGQVTVN</td><td>This work</td></tr>
<tr><td>ST-A3</td><td>CCATGGGCAGCAGCCATCATCATCATCAT<br>CACAGCAGCGGCCTGGTGCCGCGCGGCAG<br>CCACGTGCCTACTATCGTGATGGTGGACG<br>CCTACAAGCGTTACAAGAGCGATAAAATT<br>ATTGTGCTGACTGACGACAGTTTTGACAC<br>GGATGTACTCAAAGCGGACGGGGCGATCC<br>TCGTCGATTTCAACGCAGAGCAGTGCGGT<br>CCGTGCAAAATGATCGCCCCGATTCTGGA<br>TGAAATCGCTGACGAATATCAGGGCAAAC<br>TGACCGTTGCAAAACTGAACATCGATCAA<br>AACCCTGGCACTGCGCCGAAATATGGCAT<br>CCGTGGTATCCCGACTCTGCTGCTGTTCA<br>AAAACGGTGAAGTGGCGGCAACCAAAGTG<br>GGTGCACTGTCTAAAGGTCAGTTGAAAGA<br>GTTCCTCGACGCTAACCTGGCGTAAAAGC<br>TT</td><td>MGSSHHHHHHSSGLVPR|GSHVPTIVMVDAY<br>KRYKSDKIIVLTDDSFDTDVLKADGAILVDF<br>NAEQCGPCKMIAPILDEIADEYQGKLTVAKL<br>NIDQNPGTAPKYGIRGIPTLLLFKNGEVAAT<br>KVGALSKGQLKEFLDANLA</td><td>This work</td></tr>
<tr><td>SC002</td><td></td><td>VTTLSGLSGEQGPSGDMTTEEDSATHIKFSK<br>RDEDGRELAGATMELRDSSGKTISTWISDGH<br>VKDFYLYPGKYTFVETAAPDGYEVATAITFT<br>VNEQGQVTVN</td><td>[38,58]</td></tr>
<tr><td>ST002</td><td></td><td>VPTIVMVDAYKRYK</td><td>[38,58]</td></tr>
</table>

## References


[38] S. C. Reddington, M. Howarth, Curr Opin Chem Biol 2015, 29, 94–99.

[53] P. Gonzalez, K. Bossak, E. Stefaniak, C. Hureau, L. Raibaut, W. Bal, P. Faller, Chemistry - A European Journal 2018, 24, 8029–8041.

[54] P. Mlynarz, D. Valensin, K. Kociolek, J. Zabrocki, J. Olejnik, H. Kozlowski, New Journal of Chemistry 2002, 26, 264–268.

[58] L. Li, J. O. Fierer, T. A. Rapoport, M. Howarth, J Mol Biol 2014, 426, 309–317.

[59] M. Perinelli, R. Guerrini, V. Albanese, N. Marchetti, D. Bellotti, S. Gentili, M. Tegoni, M. Remelli, J Inorg Biochem 2020, 205, 110980.

[62] L. Alderighi, P. Gans, A. Ienco, D. Peters, A. Sabatini, A. Vacca, Coord Chem Rev 1999, 184, 311–318.

[72] V. Borghesani, Journal of Peptide Science 2024, n/a, e3649.

[73] K. J. McKnelly, W. Sokol, J. S. Nowick, J Org Chem 2020, 85, 1764–1768.

[74] N. A. Sole, G. Barany, J Org Chem 1992, 57, 5399–5403.

[75] C. N. Pace, F. Vajdos, L. Fee, G. Grimsley, T. Gray, Protein Science 1995, 4, 2411–2423.

[76] C. N. Pace, F. X. Schmid, in Protein Structure : A Practical Approach (Ed.: T.E. Creighton), Oxford University Press, New York, 1997.

[77] G. R. Grimsley, C. N. Pace, Curr Protoc Protein Sci 2003, 33, DOI 10.1002/0471140864.ps0301s33.

[78] A. I. Vogel, Qunatitative Inorganic Analysis Including Elementary Instrumental Analysis, Longmans, London, UK, 1962.

[79] P. Gans, A. Sabatini, A. Vacca, Talanta 1996, 43, 1739–1753.

[80] P. Gans, A. Sabatini, A. Vacca, Ann Chim 1999, 89, 45–49.

[81] M. Sokołowska, W. Bal, J Inorg Biochem 2005, 99, 1653–60.

[82] J. Jumper, R. Evans, A. Pritzel, T. Green, M. Figurnov, O. Ronneberger, K. Tunyasuvunakool, R. Bates, A. Žídek, A. Potapenko, A. Bridgland, C. Meyer, S. A. A. Kohl, A. J. Ballard, A. Cowie, B. Romera-Paredes, S. Nikolov, R. Jain, J. Adler, T. Back, S. Petersen, D. Reiman, E. Clancy, M. Zielinski, M. Steinegger, M. Pacholska, T. Berghammer, S. Bodenstein, D. Silver, O. Vinyals, A. W. Senior, K. Kavukcuoglu, P. Kohli, D. Hassabis, Nature 2021, 596, 583–589.

[83] Schrödinger, LLC, The {PyMOL} Molecular Graphics System, Version~1.8, 2015.
